# Medicaid expansion and variability in mortality in the USA: a national, observational cohort study

**DOI:** 10.1016/S2468-2667(21)00252-8

**Published:** 2021-12-02

**Authors:** Brian P Lee, Jennifer L Dodge, Norah A Terrault

**Affiliations:** Division of Gastroenterology and Liver Diseases (B P Lee MD, J L Dodge MPH, Prof N A Terrault MD) and Department of Population and Public Health Services (J L Dodge), University of Southern California Keck School of Medicine, Los Angeles, CA, USA

## Abstract

**Background:**

The expansion of the Medicaid public health insurance programme has varied by state in the USA. Longer-term mortality and factors associated with variability in outcomes after Medicaid expansion are under-studied. We aimed to investigate the association of state Medicaid expansion with all-cause mortality.

**Methods:**

This was a population-based, national, observational cohort study capturing all reported deaths among adults aged 25–64 years via death certificate data in the Centers for Disease Control and Prevention’s Wide-ranging Online Data for Epidemiologic Research (CDC WONDER) database in the USA from Jan 1, 2010, to Dec 31, 2018. We obtained national demographic and mortality data for adults aged 25–64 years, and state-level demographics and 2010–18 mortality estimates for the overall population by linking federally maintained registries (CDC WONDER, Behavioral Risk Factor Surveillance System, Health Resources and Services Administration, US Census Bureau, and Bureau of Labor Statistics). States were categorised as Medicaid expansion or non-expansion states as classified by the Kaiser Family Foundation. Multivariable difference-in-differences analysis assessed the absolute difference in the annual, state-level, all-cause mortality per 100 000 adults after Medicaid expansion.

**Findings:**

Among 32 expansion states and 17 non-expansion states, Medicaid expansion was associated with reductions in all-cause mortality (−11·8 deaths per 100 000 adults [95% CI −21·3 to −2·2]). There was variability in changes in all-cause mortality associated with Medicaid expansion by state (ranging from −63·8 deaths per 100 000 adults [95% CI −134·1 to −42·9] in Delaware to 30·4 deaths per 100 000 adults [−39·8 to 51·4] in New Mexico). State-level proportions of women (−17·8 deaths per 100 000 adults [95% CI −26·7 to −8·8] for each percentage point increase in women residents) and non-Hispanic Black residents (−1·4 deaths per 100 000 adults [−2·4 to −0·3] for each percentage point increase in non-Hispanic Black residents) were associated with greater adjusted reductions in all-cause mortality among expansion states.

**Interpretation:**

After 4 years of implementation, Medicaid expansion remains associated with significant reductions in all-cause mortality, but reductions are variable by state characteristics. These results could inform policy makers to provide broad-based equitable improvements in health outcomes.

## Introduction

Medicaid is a public health insurance programme and the largest provider of health insurance in the USA, covering more than 75 million people. Medicaid policies are instituted at the state level and are intended to provide health coverage for vulnerable populations, including low-income individuals and people with disabilities. The Affordable Care Act (ACA) was federal legislation taking effect in 2014, which provided the option to states to expand Medicaid to individuals not previously eligible, increasing the income qualification to 138% of the federal poverty level.^1^ Although originally mandated nationwide, the US Supreme Court later ruled that expansion must be optional for states. Medicaid expansion provided health insurance for an additional 12 million people, accounting for the majority of the increase in insurance coverage resultant from the ACA.^1^ Some states have chosen not to expand Medicaid and the future of Medicaid expansion is still debated.^2^

Objective and quantitative data regarding the granular ramifications of Medicaid expansion can inform policy interventions involving public health insurance. Although previous studies have reported overall population-level improvement in health-related outcomes,^3–5^ including all-cause mortality and within specific disease groups,^6,7^ full national outcomes studies have been limited to insurance eligibility, or 1-year or 2-year mortality outcomes following Medicaid expansion, and none have provided in-depth analyses on potential state-level variability.^8–11^ Additionally, the lag-time of potential effects of Medicaid expansion might not yet be apparent in these previous reports as the majority of adult mortality can be linked to chronic medical conditions. For example, the lag-time to benefit of statins to reduce cardiac events is 2·5 years, and of breast and colorectal cancer screening to reduce neoplasia-related mortality are 3·0 years and 4·8 years, respectively.^12,13^ The most contemporary and complete appraisal of available data should be used to inform impending policy decisions.

Variability and underlying reasons for heterogeneous effects of Medicaid expansion (eg, medical condition case mix and geographical characteristics) on health outcomes are underexplored, but are relevant for policy makers in identifying medical conditions and geographical areas that would be most affected by policy interventions to Medicaid coverage. Some states have expanded Medicaid, whereas some have not—we sought to use this natural experiment to determine the association of state Medicaid expansion with all-cause mortality, and carry out exploratory analyses to elucidate more granular associations of Medicaid expansion with cause-specific mortality to inform ongoing policy interventions, both within the USA and other countries considering changes to public health coverage options.

## Methods

### Study design and participants

This was a population-based, national, observational cohort study capturing all reported deaths among adults aged 25–64 years via death certificate data in the Centers for Disease Control and Prevention’s Wide-ranging Online Data for Epidemiologic Research (CDC WONDER) database in the USA from Jan 1, 2010, to Dec 31, 2018 (appendix p 3). This study was considered exempt from Institutional Review Board approval as a deidentified public national database study.

### Time-varying covariates

State-level characteristics that we a-priori hypothesised could be confounders or mediators of mortality were obtained from national registries. We modelled these characteristics as time-varying covariates by year, linked at the closest year of available data. From the US Census Bureau, we obtained the state-level proportion of residents aged 25–64 years who were female, and the proportions who were non-Hispanic Black, Hispanic, non-Hispanic White, or Asian; the proportion of residents in each 10-year age group between 18 and 64 years without health insurance; and for the overall population, median household income and the proportion living in poverty. From the US Bureau of Labor Statistics, we obtained the unemployment rate. From the Behavioral Risk Factor Surveillance System, we obtained the proportion of adults with diabetes, obesity, currently smoking, reporting any alcohol use in the past 30 days, binge drinking in the past 30 days, self-reported good or better health, and routine medical care in the past 5 years. From the Health Resources and Services Administration, we obtained the number of primary care physicians per 100 000 population, total hospital beds per 1000 population, and the proportion of residents living in rural areas. The full variable list with years of data availability is shown in the appendix (p 4).

### Categorisation of exposure variable and study period

For the exposure variable, Medicaid expansion status, states were categorised as expansion or non-expansion as classified by the Kaiser Family Foundation. The majority of expansion states (n=25) implemented Medicaid expansion on Jan 1, 2014, including six states (California, Connecticut, District of Columbia, Minnesota, New Jersey, and Washington) with early (before 2014 but after ACA passage in 2010) limited expansion before full Medicaid expansion. Seven states (Michigan, New Hampshire, Pennsylvania, Indiana, Alaska, Montana, and Louisiana) had late expansion (after Jan 1, 2014). Early and late expansion states were categorised as expansion in the main analyses but excluded from sensitivity analyses. Four states (Virginia, Utah, Maine, and Idaho) implemented expansion in 2019–20, but were categorised as non-expansion, given the study period was 2010–18 (most recent availability of CDC WONDER estimates for this analysis). Massachusetts and Wisconsin had previous Medicaid expansion independent of ACA and were excluded from analyses. Expansion dates and categorisation are shown in the appendix (p 5).

### Outcomes

The primary outcome was the annual, state-level, all-cause mortality per 100 000 adults. We extracted age-adjusted mortality rates by state of residence and year of death from 2010 to 2018 in 10-year age groups (25–34, 35–44, 45–54, and 55–64 years) to specifically capture the Medicaid-eligible population. Mortality was standardised to the 2000 US standard population using the direct method.

In prespecified exploratory analysis of cause-specific mortality, external (ie, injuries, suicide, homicide, medical treatment complications, and substance use) versus internal (ie, non-external) causes of death were evaluated by International Classification of Diseases version 10 (ICD-10) codes, as in other studies.^3^ We also evaluated cardiovascular-related, respiratory-related, neoplasia-related, prescription or opioid overdose-related, and infection-related mortality. We defined deaths attributable to prescription or opioid overdose as they are defined in the ICD-10 manual of drug poisoning (similarly to the CDC^14^), which includes illicit drug poisoning and prescription opioid poisoning (appendix p 6). We chose these subcategories of common medical conditions hypothesising that patients with chronic diseases (eg, cardiovascular and respiratory conditions) would have a greater benefit from access to preventive care offered through Medicaid expansion, compared with patients with conditions with greater relative acuity (prescription or opioid overdoses, infections, and neoplasia).

### Statistical analysis

The sample size was fixed and used the entirety of each state’s population, rather than a sample; therefore, estimates reflect the whole population and sample size and power were not calculated as the study size has full national case ascertainment. CIs are provided to guide interpretation of results.

State-level characteristics were described by Medicaid expansion status as mean (SD) and n (%), weighted by the state population aged 25–64 years, and compared using *t* tests and χ² tests. Annual state-level, age-adjusted mortality was modelled as the dependent variable using linear regression to calculate the mean age-adjusted mortality among expansion and non-expansion states. To address within-state correlation in repeated measures by year, the models accounted for clustering by state and used standard errors based on Huber-White sandwich estimators, which are robust to heteroscedasticity and autocorrelation. Mortality was then adjusted for the proportion of the state population in each 10-year age group (35–44, 45–54, and 55–64 years), the proportion who were female, the proportion who were non-Hispanic Black or Hispanic, the proportion living in poverty, and the proportion who were unemployed using multivariable linear regression. Our a-priori hypothesis was that other covariates were correlated with or mediators of Medicaid expansion (ie, proportion uninsured, median income, and number of physicians and hospital beds per capita) and were not included in the primary multivariable model. To evaluate the association of Medicaid expansion with mortality trends, the exposure (Medicaid expansion status) and era (pre-expansion *vs* post-expansion), along with an interaction term between the two (overall difference-in-differences estimator), were added to the model. All-cause and cause-specific mortality were stimated in separate models. All models were weighted to the state population aged 25–64 years.

State-specific difference-in-differences were estimated separately for each expansion state compared with the sample of non-expansion states adjusted for the main model covariates. As clustered inference issues might arise with only one treated cluster, we calculated 95% CIs using the population-weighted linear probability approach developed by Conley and Taber^15^ and applied a Holm step-down corrected significance level to address multiple comparisons.^16^ To test the hypothesis that state-level heterogeneity in the effect of Medicaid expansion was associated with the demographic makeup of states, the resulting state-specific difference-indifferences for all cause-mortality were modelled as the outcome using linear regression and assessed for associations with 2014 state demographics (proportion who were female, proportion who were non-Hispanic Black or Hispanic, proportion who were rural residents, and change in proportion who were uninsured from 2014 to 2018) adjusting for baseline mortality.

The difference-in-differences assumption of parallel trends was evaluated pre-Medicaid expansion (2010–13) for all-cause and cause-specific mortality and for each expansion state separately using an interaction term between Medicaid expansion status and year as a continuous linear variable (appendix pp 7–9). States violating the parallel trends assumption were excluded from the state-specific difference-in-differences analysis.

In a prespecified exploratory analysis to inform generalisability of our findings, we sought to assess potential differential effects in the association of changes in the uninsured population with mortality among expansion versus non-expansion states. We modelled absolute change from 2010 to 2018 in all-cause mortality (outcome) and state-level proportion of uninsured residents (explanatory variable) using linear regression adjusting for baseline age-adjusted mortality. We tested the interaction between insurance change and expansion status to determine if the effect of a decrease in uninsured residents differed by Medicaid expansion status.

We carried out sensitivity analyses to assess the robustness of our results. We repeated the all-cause mortality analysis at the county level to assess for potential bias due to county-level variability within states. Counties with nine or fewer deaths per year are suppressed in the CDC WONDER database for county-level analyses; therefore, the number of included counties varied by year from 2475 (79%) of 3143 counties (or county equivalents) in 2010 to 2513 (80%) in 2018 in this sensitivity analysis (no counties were suppressed in the main state-level analysis). To assess for confounding by staggered timing of Medicaid expansion, we did two analyses excluding states with limited early expansion (n=6) and excluding states with late expansion (n=7). To assess for confounding from concomitant expanded access to health insurance not through Medicaid expansion, we did a sensitivity analysis adjusting for the proportion of the population without health insurance. We hypothesised that changes in income could be a mediator of Medicaid expansion-related improved health outcomes over time, so income was excluded from the main model; however, we did a sensitivity analysis adjusting for median household income (inflation-adjusted to 2018 US dollars) under the assumption that income was a confounder, and not a mediator.

We repeated the state-specific all-cause mortality analysis using an interrupted time-series approach to confirm the difference-in-differences results. Briefly, we compared each Medicaid expansion state to the control group of non-expansion states using segmented ordinary least-squares regression with Newey-West standard errors to account for autocorrelation (Stata Interrupted Time-Series Analysis command). Models remained weighted, accounted for clustering, and included covariates as in the main analysis. We report the effect of Medicaid expansion on mortality as the interaction between Medicaid expansion status and era (pre-expansion *vs* post-expansion).

p values are from two-sided hypothesis tests in which values less than 0·05 indicate statistical significance, except in state-level analyses in which corrected significance levels for multiple comparisons were applied. All data analyses were carried out using SAS (version 9.4) or Stata/MP (version 16.1).

### Role of the funding source

The funder of the study had no role in study design, data collection, data analysis, data interpretation, or writing of the report.

## Results

The study included 31 expansion states and the District of Columbia, and 17 non-expansion states. At baseline in 2010, expansion states had smaller proportions of people from minority racial and ethnic groups, smaller rural populations, and fewer uninsured adults than non-expansion states (table 1). Between baseline and the end of the study period in 2018, the decrease in the proportion of uninsured adults was greater in expansion states (−10·5% absolute change; −51·9% relative change) than in non-expansion states (−7·7% absolute change; −30·3% relative change).

**Figure 1:**
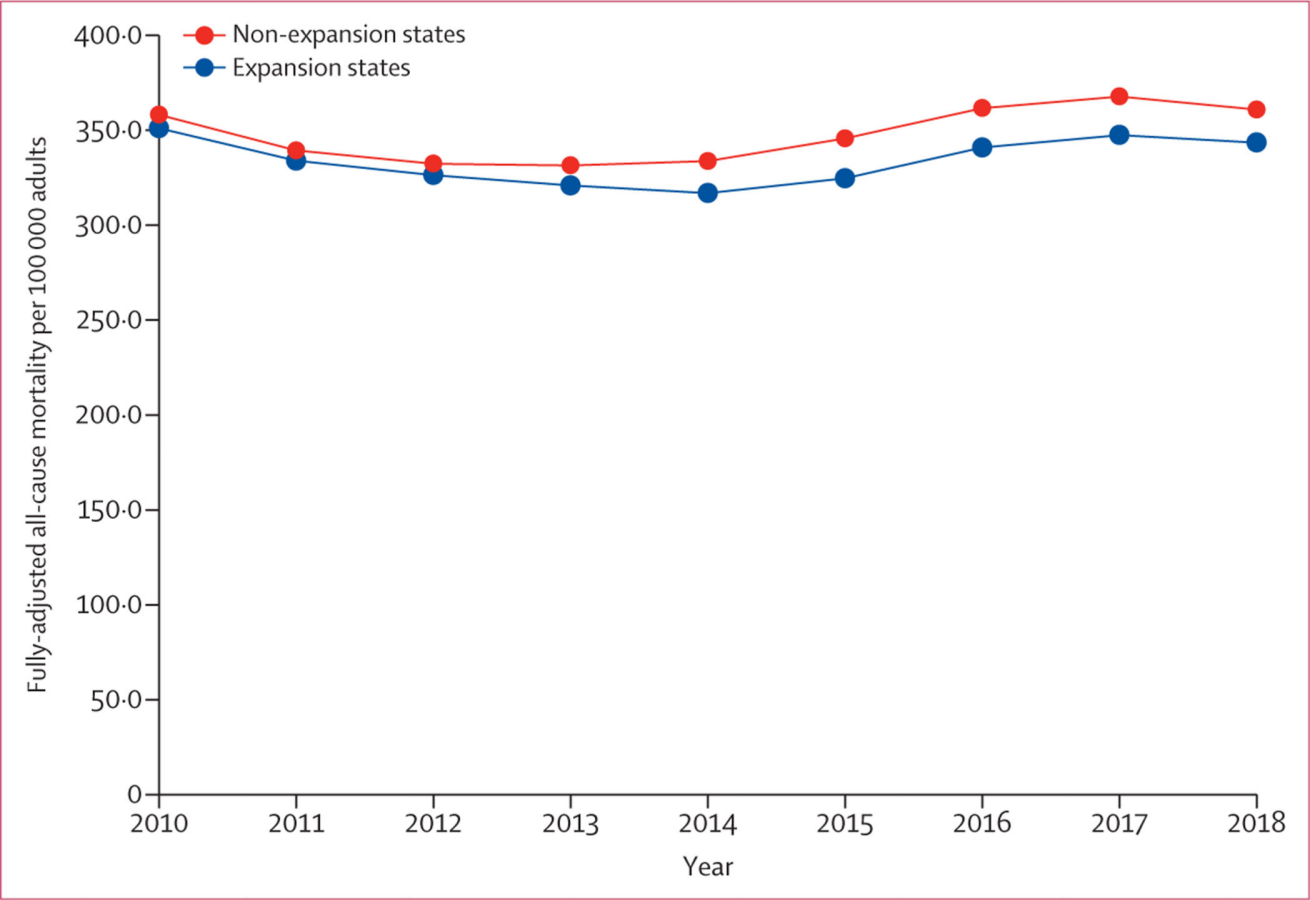
Fully adjusted all-cause mortality in expansion versus non-expansion states All-cause mortality per 100 000 adults by year, adjusted for age strata, proportion female, proportion non-Hispanic Black, proportion Hispanic, proportion in poverty, and proportion unemployed.

Baseline age-adjusted all-cause mortality was 311·2 per 100 000 adults in expansion states versus 365·2 per 100 000 adults in non-expansion states. Fully adjusted all-cause mortality from baseline to the end of the study period is shown in figure 1. In multivariable difference-in-differences analysis, expansion states had 11·8 fewer deaths per 100 000 adults per year (95% CI 2·2–21·3) from any cause after Medicaid expansion than in non-expansion states.

**Figure 2:**
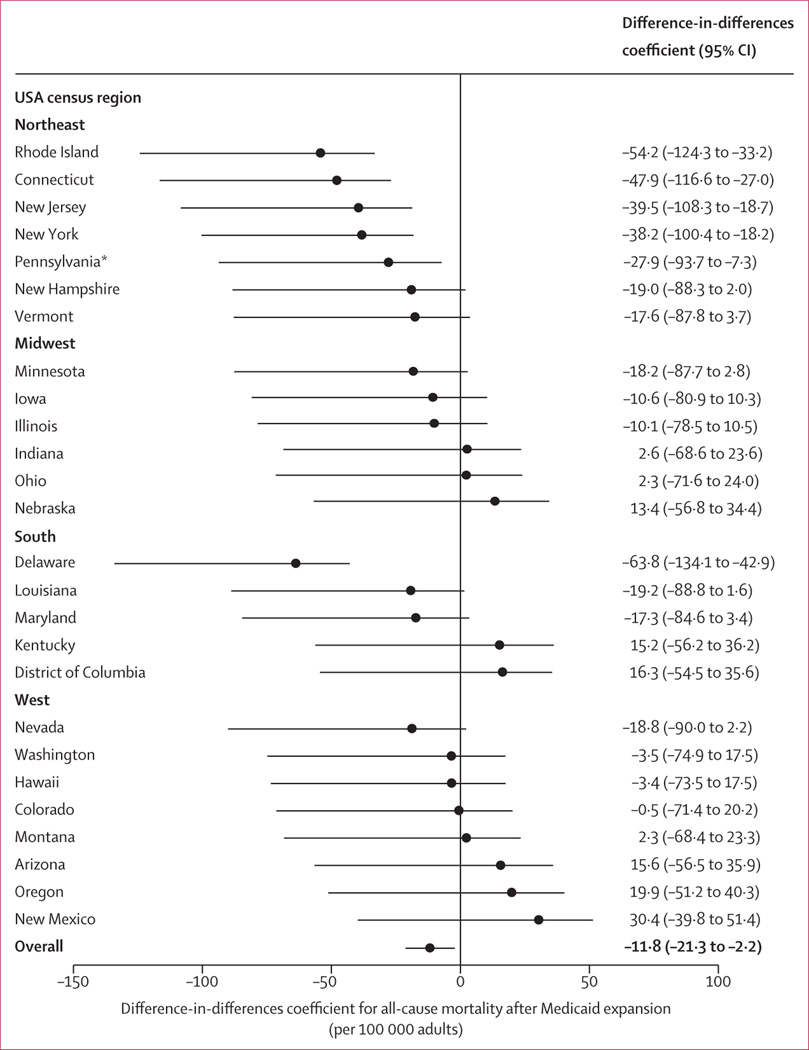
Post-expansion differences in all-cause mortality among expansion states The adjusted absolute difference in all-cause mortality per 100 000 adults after Medicaid expansion with Conley and Taber ^15^ 95% CIs obtained through multivariable difference-in-differences analysis, are shown for each expansion state. Negative values represent reductions in all-cause mortality, whereas positive values represent increases in all-cause mortality. State-specific difference-in-differences were estimated separately for each expansion state compared with the sample of non-expansion states. Alaska, Arkansas, California, Michigan, North Dakota, and West Virginia were excluded as they violated the parallel trends assumption. Delaware, Rhode Island, Connecticut, New Jersey, New York, and Pennsylvania had post-expansion differences with Conley and Taber^15^ 95% CIs that did not cross 1. *Pennsylvania did not meet the Holm step-down threshold^16^ for statistical significance.

There was variability among states in the reduction in all-cause mortality associated with Medicaid expansion. In multivariable difference-in-differences, Delaware and Rhode Island had the greatest reductions in mortality, whereas New Mexico had increased all-cause mortality associated with Medicaid expansion (figure 2). However, positive-in-sign point estimates (ie, increases in all-cause mortality associated with Medicaid expansion) were not statistically significant after correcting for multiple comparisons (figure 2; appendix p 10).

State demographic characteristics associated with reductions in difference-in-differences for all-cause mortality were the proportion of women residents, the proportion of non-Hispanic Black residents, and the change in the proportion of the population who were uninsured. From bivariate linear regression adjusting for baseline age-adjusted mortality, there were 1·4 fewer deaths per 100 000 adults (95% CI 0·3–2·4) for each percentage point increase in non-Hispanic Black residents, and 17·8 fewer deaths per 100 000 residents (8·8–26·7) for each percentage point increase in women residents. In a separate bivariate linear regression adjusting for baseline age-adjusted mortality, there were 5·4 fewer deaths per 100 000 adults (1·8–9·0) for each percentage point decrease in the state proportion of uninsured residents from 2014 to 2018. The proportion of Hispanic residents and the proportion of rural residents were not significantly associated with state-level difference-in-differences for all-cause mortality (appendix p 11).

In our exploratory analysis including non-expansion states, there were 1·6 fewer deaths per 100 000 adults (95% CI 0·2–3·0) for each percentage point decrease in state proportion of uninsured residents. There was not a statistically significant interaction of state expansion status with change in state proportion of uninsured residents (p=0·38), which suggests that the association of decrease in uninsured residents with reduction in all-cause mortality is independent of Medicaid expansion status.

The results of the exploratory analysis of fully adjusted mortality, including cardiovascular-related, respiratory-related, neoplasia-related, prescription or opioid overdose-related, and infection-related mortality for expansion versus non-expansion states are shown in table 2 and the appendix (pp 19–22). In multivariable difference-in-differences, compared with non-expansion, Medicaid expansion was associated with fewer cardiovascular-related and respiratory-related deaths per 100 000 adults per year and fewer deaths with internal causes per 100 000 adults per year (table 2). There was no difference between Medicaid expansion and non-expansion in neoplasia-related, prescription or opioid overdose-related, or infection-related deaths per 100 000 adults per year, or in the number of deaths with external causes per 100 000 adults per year (table 2).

In the state-specific difference-in-differences analyses, states with a reduction in all-cause deaths associated with Medicaid expansion (eg, Delaware, Connecticut, Maryland, New Jersey, and New Hampshire) generally had a reduction in cardiovascular-related, respiratory-related, neoplasia-related, and infection-related deaths; states with an increase in all-cause deaths associated with Medicaid expansion (eg, Nebraska, New Mexico, and Oregon) generally had an increase in the same cause-specific deaths (appendix pp 23–26). However, some states with a reduction in all-cause deaths associated with Medicaid expansion observed an increase in prescription or opioid overdose-related deaths, and some states with an increase in all-cause deaths associated with Medicaid expansion observed a reduction in prescription or opioid overdose-related deaths (appendix p 27).

Results of the sensitivity analysis done at the county level (rather than state level), and analyses excluding states that were early adopters of Medicaid expansion (n=6) and excluding states that were late adopters of Medicaid expansion (n=7) are shown in the appendix (pp 12–14). Results of the sensitivity analysis adjusting for the proportion of the population without health insurance and adjusting for state median income are shown in the appendix (pp 15–16). Results for sensitivity analyses of cause-specific mortality are summarised in the appendix (pp 12–16). The sensitivity analysis using interrupted time-series analysis for all-cause mortality for each Medicaid expansion state compared with the control group of non-expansion states yielded similar coefficients as difference-in-differences analysis with overlapping 95% CIs (appendix p 10).

## Discussion

In this study, we provide a contemporary appraisal of the association of Medicaid expansion with mortality in the USA. We show that Medicaid expansion has been associated with significant reductions in all-cause mortality, but the effects have been heterogeneous by state and cause-specific mortality. We show that the reduction in all-cause mortality from Medicaid expansion was associated with a state-level reduction in the proportion of the population who were uninsured, and that this association was also present in the analysis including non-expansion states. These findings support the hypothesis that the main mechanism by which Medicaid expansion is likely to lead to improvements in mortality is through gains in insurance coverage, as our study shows that the magnitude of change in the proportion of people who are uninsured is associated with the magnitude of improvement in overall mortality attributable to Medicaid expansion. This finding supports consideration of generalisability of Medicaid expansion to other states and highlights the potential public health benefits of expanding access to public health insurance.

We noted variability by state in the reduction in all-cause mortality associated with Medicaid expansion. We examined state-level characteristics associated with these reductions, identifying a greater proportion of women and non-Hispanic Black residents to be associated with reductions in all-cause mortality associated with Medicaid expansion. These findings suggest that women and non-Hispanic Black residents might have benefited most from Medicaid expansion. Women and non-Hispanic Black individuals have greater rates of poverty than men and individuals from other ethnic back grounds.^17^ Additionally, previous studies show that even after controlling for socioeconomic conditions, including employment, women and Black individuals have lower rates of health coverage and higher premiums for insurance plans than men and individuals of other races.^17^ Also, Black individuals and women on average have more dependents in the household, and we hypothesise that these individuals might be more motivated to obtain insurance when provided with the option. Thus, our results suggest that interventions to Medicaid policy might have especially pronounced effects on racial and sex differences in health outcomes, which need careful consideration by policy makers. Also, as non-expansion (*vs* expansion) states had a higher proportion of Black individuals, and the association between reduction in uninsured residents with decreases in all-cause mortality was independent of Medicaid expansion status, our analyses suggest that non-expansion states might have the most to gain from adopting Medicaid expansion.

We also assessed cause-specific mortality as a potential contributor to variability in the effects of Medicaid expansion, which showed significant reductions associated with Medicaid expansion in internal causes, cardiovascular-related and respiratory-related mortality, but not for other causes we examined. We hypothesise these findings to be secondary to greater access to preventive care, specialist referrals, and medications, among other services provided by Medicaid. Given that cardiovascular-related and respiratory-related mortality accounts for more than a third^18^ of national deaths in the USA, these results are impressive, particularly given the relatively short time that Medicaid expansion has been in place.

Prescription or opioid overdose-related deaths showed some discordant state-specific trends compared with other causes of death examined. States with the greatest reductions in all-cause mortality (eg, Delaware, New Jersey, and New Hampshire) generally had the greatest reductions in cardiovascular-related, respiratory-related, neoplasia-related, and infection-related deaths, but not in prescription or opioid overdose-related deaths. Indeed, our results show prescription or opioid overdose- related deaths more than doubled between 2010 and 2018 among expansion states, more than the 61% relative increase among non-expansion states, although this was not statistically significant. Treatment financing for specific opioid use disorder services is not fully guaranteed by Medicaid and varies by state—previous studies show 80% of individuals with opioid use disorder do not receive active treatment—and there has been concern that Medicaid expansion might be accompanied by declines in state-level spending on opioid use disorder treatment.^19^ Additionally, a recent national study showed that Medicaid expansion was associated with a reduction in deaths from heroin and synthetic opioids, but an increase in methadone-related overdose—this increase was hypothesised to be related to high rates of methadone used to treat pain and disproportionately associated with overdose deaths among individuals using Medicaid.^20^ Whether Medicaid expansion con tributes to increased opioid deaths through increased access to prescription pain medication needs further evaluation. Our results high light diseases that might require targeted policy interventions to ensure that not only insurance gaps are addressed, but treatment gaps as well, to provide more equitable and broad improvements in health outcomes.

Medicaid expansion, which increased access to health insurance for individuals with low income, might potentially be a policy intervention to bridge disparities across the wealth spectrum. In the USA, the wealthiest 1% of women live 10 years longer than the poorest 1% of women, and the wealthiest 1% of men live 14 years longer than the poorest 1% of men.^21^ Medical debt is present among 18% of US adults, and is higher among people with low incomes than those with high incomes, and among those without health insurance, which might be alleviated by Medicaid expansion.^22^ A recent national study showed that Medicaid expansion was associated with a 34% absolute difference in decline in medical debt, compared with states without Medicaid expansion.^22^ Indeed, our main results were attenuated by adjustment for time-varying median income, which suggests that the association between Medicaid expansion and mortality outcomes might be mediated by changes in income. Income interventions for low-income populations, in the form of tax credits, have had positive effects on health outcomes, particularly among minority racial and ethnic groups and women.^21^ These findings, supplemented by our results showing the most substantial effects of Medicaid expansion among states with a higher proportion of Black individuals, highlight the potential pleiotropic effects of expanded access to health-care coverage to affect health and financial wellbeing for vulnerable populations.

This study has limitations. First, the data are observational and aggregated, so residual confounding and individual-level characteristics affecting estimates are possible. For example, Medicaid expansion (or choosing not to expand) could be associated with other health-related state-level changes, and could influence our estimates regarding the effects of Medicaid expansion for specific demographics and states. However, we provide several analytical approaches to address these limitations, including difference-in-differences to adjust for secular trends, county-level sensitivity analyses to adjust for more granular county-level variability, and adjustment for time-varying covariates, which is distinct from previous Medicaid expansion studies that largely adjusted for fixed baseline covariates. Second, different timing of Medicaid expansion among states and lag-time of exposure effect, especially for chronic diseases, might introduce bias. However, these limitations would likely bias results towards the null, and results from sensitivity analyses excluding states with different timing of Medicaid expansion did not substantially differ from the primary estimates. Third, the data are aggregated, so the covariates assessed (eg, the proportion of women) are at the state level and might not reflect individual-level associations. Finally, the CDC WONDER data are based on death certificates, focusing on a single underlying cause of death by ICD-10 code, so misclassification of cause-specific mortality is possible and analysis of cause-specific mortality should be considered an exploratory outcome. Data are expected to be 100% complete for all-cause mortality; however; bias from death certificate data in specifying underlying cause of death is possible.

In conclusion, our study provides contemporaneous results regarding the association of Medicaid expansion with mortality across the USA, and novel insight into the variability of this association. Policy makers might find such results helpful to inform potential ramifications of impending policy decisions, and for future policies aiming to provide broad-based equitable improvements in health outcomes.

## Supplementary Material

1

## Figures and Tables

**Table 1:** Baseline state characteristics by Medicaid expansion status in 2010

	Expansion states (n=32)	Non-expansion states (n=17)

Women	50·5% (0·6)	50·8% (0·5)
Non-Hispanic Black	9·9% (6·7)	16·4% (8·0)
Non-Hispanic White	66·0% (16·0)	63·3% (11·8)
Hispanic	15·2% (11·7)	15·5% (12·1)
Asian	6·8% (5·6)	3·1% (1·4)
Lives in rural county	16·6% (11·1)	23·9% (11·7)
Without health insurance	19·9% (4·4)	25·7% (5·0)
Without health insurance with income <138% of the federal poverty line	38·1% (6·8)	48·1% (6·9)
US census region		
Northeast	7 (25·6%)	1 (1·3%)
Midwest	8 (27·3%)	3 (8·7%)
South	7 (11·2%)	10 (85·8%)
West	10 (35·9%)	3 (4·2%)
Unemployed	9·9% (1·9)	9·4% (1·5)
In poverty	14·7% (2·4)	16·7% (2·3)
Household income, US$	$54 047 (46 536–57 664)	$44 390 (43 417–48 622)
Primary care physicians per 100 000 residents	30·0 (8·5)	28·6 (4·9)
Hospital beds per 1000 residents	3·0 (0·7)	3·3 (0·6)
Underlying medical conditions		
Diabetes	8·9% (1·1)	9·9% (1·2)
Obesity	26·9% (2·8)	29·9% (2·5)
Current smoking	16·6% (3·6)	18·1% (2·7)
Any alcohol in past 30 days	54·7% (5·4)	47·0% (7·9)
Binge alcohol in past 30 days	15·5% (1·8)	13·1% (2·5)
Self-reported good or better health	84·3% (2·6)	82·8% (2·3)
Ever myocardial infarction or coronary heart disease	4·1% (0·7)	4·6% (0·7)
Ever stroke	2·6% (0·5)	3·2% (0·6)
Asthma	9·0% (1·1)	7·9% (0·8)

Data are mean (SD), n (%), or median (IQR). Mean (SD) and percentages are weighted by the state population aged 25–64 years.

**Table 2:** Difference-in-differences analysis for subcategories of causes of mortality

	Adjusted mortality in expansion states in 2010 per 100 000 adults	Absolute difference* in mortality after Medicaid expansion per 100 000 adults per year (95% CI)

All causes	351·1	−11·8 (−21·3 to −2·2)
Internal causes	274·6	−11·2 (−17·8 to −4·7)
External causes	76·6	−0·5 (−5·9 to 4·9)
Cardiovascular	74·7	−5·0 (−7·4 to −2·7)
Respiratory	16·3	−1·6 (−2·7 to −0·5)
Neoplasia	91·2	−0·9 (−2·4 to 0·7)
Infection	16·8	−0·2 (−1·2 to 0·8)
Prescription or opioid overdose	13·5	2·7 (−1·2 to 6·6)

* Difference-in-differences analysis, which adjusted for age strata, proportion female, proportion non-Hispanic Black, proportion Hispanic, proportion in poverty, proportion unemployed, and the before–after trends in non-expansion states.

## Research in context

### Evidence before this study

Medicaid is a government health insurance programme intended to provide coverage for vulnerable populations, including low-income individuals and people with disabilities, and serves as the largest provider of health insurance in the USA. In 2014, federal legislation expanded Medicaid eligibility, but adoption of Medicaid expansion has varied by state. We searched PubMed with no language restrictions from inception to June 1, 2021, using the following terms: “(Medicaid AND expansion AND mortality)”. Previous studies show improvement in 1-year or 2-year mortality outcomes following Medicaid expansion. None have provided in-depth analyses of potential state-level variability in outcomes. Longer-term mortality outcomes and factors associated with variability in health outcomes are under-studied.

### Added value of this study

Our results with longer-term follow-up show that Medicaid expansion is associated with significant reductions in all-cause mortality. We show that there is variability in the reductions by state characteristics and cause-specific mortality. State-level decreases in the proportion of people who are uninsured and higher proportions of non-Hispanic Black individuals and women were associated with greater reductions in all-cause mortality associated with Medicaid expansion.

### Implications of all the available evidence

These findings suggest that Medicaid expansion reduces overall mortality, mediated by gains in insurance coverage. These results could inform policy makers, government officials, and health-care systems regarding the effects of public health insurance coverage on population-level mortality outcomes, particularly among countries that do not provide universal health coverage. Variability among cause-specific mortality highlights potential gaps in health services that persist even after gaining health coverage, which is informative for high-income countries with high levels of health coverage and considering changes to access in health services. Expanded health coverage might help countries address inequities based on wealth, race, and sex.
