# Supplementary material for: Medicaid expansion and variability in mortality in the USA: a national, observational cohort study
Source: Lancet Public Health. Author manuscript; Available in PMC 2023 Apr 23. (PMC10122976; doi:10.1016/S2468-2667(21)00252-8)
Supplement: 1 [file NIHMS1768916-supplement-1.pdf]

THE LANCET
Public Health

## Supplementary appendix

This appendix formed part of the original submission and has been peer reviewed. We post it as supplied by the authors.

Supplement to: Lee BP, Dodge JL, Terrault NA. Medicaid expansion and variability in mortality in the USA: a national, observational cohort study. *Lancet Public Health* 2021; published online Dec 2. https://doi.org/10.1016/S2468-2667(21)00252-8.

**Medicaid Expansion and Variability with Mortality**

**Supplementary Material**

Table of contents:

Supplementary Methods……………………………………………………….…………………………......p.3

Supplementary Tables

Supplemental Table 1. Co-variates, Data Sources, and Years of Availability …….………………………….........p.4
Supplemental Table 2. Medicaid Expansion Categorization for Primary Analyses….……………………..….........p.5
Supplemental Table 3. ICD-10 Codes for Cause-Specific Mortality……….……….………………………….......p.6
Supplemental Table 4. Evaluation of Parallel Trends Assumption for Difference-in-Differences Assessment between 2010-2013 for All-Cause and Cause-Specific Mortality ……………………………………………………........p.7
Supplemental Table 5. Evaluation of Parallel Trends Assumption for Difference-in-Differences Assessment between 2010-2013 for Cardiovascular-, Respiratory, and Neoplasia- Mortality by State………………………...….........p.8
Supplemental Table 6. Evaluation of Parallel Trends Assumption for Difference-in-Differences Assessment between 2010-2013 for Infection- and Prescription/Opioid-Related Mortality by State……………………………….......p.9
Supplemental Table 7. State-Specific Difference-in-Differences Estimates and Interrupted Time Series Analysis for All-Cause Mortality ……………………………………………………………………...………..........……p.10
Supplemental Table 8. State Characteristics Associated with Reduction in Difference-in-Differences for All-Cause Mortality Associated with Medicaid Expansion (Linear Regression)…………………………...…...........……p.11
Supplemental Table 9. Sensitivity Analysis 1: County-Level Difference-in-Differences Analysis for Subcategories of Mortality ……………………….……………………………………………………………………......p.12
Supplemental Table 10. Sensitivity Analysis 2: Difference-in-Differences Analysis for Subcategories of Mortality, Excluding Early (N=6) Adopters of Medicaid Expansion ……………………………………………...….......p.13
Supplemental Table 11. Sensitivity Analysis 2: Difference-in-Differences Analysis for Subcategories of Mortality, Excluding Late (N=7) Adopters of Medicaid Expansion ………………………………………………….........p.14
Supplemental Table 12. Sensitivity Analysis 3: Difference-in-Differences Analysis for Subcategories of Mortality, Adjusting for Proportion of Population without Health Insurance………………………………………………p.15
Supplemental Table 13. Sensitivity Analysis 4: Difference-in-Differences Analysis for Subcategories of Mortality, Adjusting for Median State Income………………..……………………………………………….……........p.16

Supplementary Figures

Supplemental Figure Legend.……………………………………..……..………………………………...…p.17
Supplemental Figure 1. Cohort Flowchart.……………………………………..………..………………...…p.18
Supplemental Figure 2. Fully-Adjusted Mortality in Expansion vs. Non-Expansion States for Internal Causes of Death ……………………..……………………...…………………………………………….….…...p.19
Supplemental Figure 3. Fully-Adjusted Mortality in Expansion vs. Non-Expansion States for External Causes of Death ……………………..…………………..……..………………………………………...……….....p.19
Supplemental Figure 4. Fully-Adjusted Mortality in Expansion vs. Non-Expansion States for Cardiovascular-Related Mortality ……………………………………..…………………………………………….…...p.20
Supplemental Figure 5. Fully-Adjusted Mortality in Expansion vs. Non-Expansion States for Respiratory-Related Mortality …………………..…………………………..…………………………………………….…...p.20
Supplemental Figure 6. Fully-Adjusted Mortality in Expansion vs. Non-Expansion States for Neoplasia-Related Mortality …………………..……………………………..………………………………………….……...p.21
Supplemental Figure 7. Fully-Adjusted Mortality in Expansion vs. Non-Expansion States for Infection-Related Mortality ……………………………………………………………………..……..…………………….…...p.21
Supplemental Figure 8. Fully-Adjusted Mortality in Expansion vs. Non-Expansion States for Prescription/Opioid Overdose Mortality …………………………………………………………………...……………………….…...p.22
Supplemental Figure 9. Post-Expansion Difference-in-Differences in Cardiovascular-Related Mortality among Expansion States……………………....…………………………………………………………………….…...p.23
Supplemental Figure 10. Post-Expansion Difference-in-Differences in Respiratory-Related Mortality among Expansion States……………………....…………………………………………………………………….…...p.24

Supplemental Figure 11. Post-Expansion Difference-in-Differences in Neoplasia-Related Mortality among Expansion States……………………....………………………………………………………………….…...p.25
Supplemental Figure 12. Post-Expansion Difference-in-Differences in Infection-Related Mortality among Expansion States……………………....………………………………………………..……………………………….…...p.26
Supplemental Figure 13. Post-Expansion Difference-in-Differences in Prescription/Opioid Overdose-Related Mortality among Expansion States……………………………………….……………...…………………….…...p.27

## SUPPLEMENTARY METHODS

### Center for Disease Control and Prevention's Wide-ranging Online Data for Epidemiologic Research (CDC WONDER)

CDC WONDER is a publicly-available database, which provides mortality and population counts for all US counties. Data are based on US death certificates, which identify a single underlying cause of death and demographic data. WONDER then uses the number of deaths as the numerator and the corresponding county-population size based on US Census Bureau data as the denominator with age-adjusted death rates calculated per 100,000 persons. Each estimate is accompanied by a 95% confidence interval and standard error. We used the Detailed Mortality File, which provides the number and rates of death aggregated by age, race, ethnicity, sex, place of death, date of death, whether an autopsy was performed, and the underlying cause of death, coded by the International Classification of Diseases (ICD)-10 system.

**Supplemental Table 1. Co-Variates, Data Sources, and Years of Availability**

| Co-Variate | Data Source | Year Available |
|---|---|---|
| Sex | US Census Bureau | All years |
| Race/ethnicity | US Census Bureau | All years |
| Poverty | US Census Bureau | All years |
| Unemployed | US Bureau of Labor Statistics | All years |
| Rural | Health Resources and Services Administration | 2010* |
| Uninsured (health insurance) | US Census Bureau | All years |
| Household income | US Census Bureau | All years |
| Diabetes | Behavioral Risk Factor Surveillance System | All years |
| Obesity | Behavioral Risk Factor Surveillance System | All years |
| Smoking | Behavioral Risk Factor Surveillance System | All years |
| Alcohol consumption | Behavioral Risk Factor Surveillance System | All years |
| Good health days | Behavioral Risk Factor Surveillance System | All years |
| Routine medical check-up | Behavioral Risk Factor Surveillance System | All years |
| Ever Myocardial Infarction or Ischemic Heart Disease | Behavioral Risk Factor Surveillance System | All years |
| Stroke | Behavioral Risk Factor Surveillance System | All years |
| Asthma | Behavioral Risk Factor Surveillance System | All years |
| Primary care physicians | Health Resources and Services Administration | 2010, 2015, 2017* |
| Cardiologists | Health Resources and Services Administration | 2010, 2015, 2017* |
| Hospital beds per capita | Health Resources and Services Administration | 2010, 2015, 2017* |

*For co-variates in which all years were not available, the 2010 value was applied to years 2010-2014, the 2015 value was applied to 2015-2016, and the 2017 value was applied to 2017-2018. For the rural co-variate, 2010 was applied to all years.

**Supplemental Table 2. Medicaid Expansion Categorization for Primary Analyses**

| Expansion* | Non-Expansion |
|---|---|
| Alaska*** | Alabama |
| Arizona | Florida |
| Arkansas | Georgia |
| California** | Idaho |
| Colorado | Kansas |
| Connecticut** | Maine |
| DC** | Mississippi |
| Delaware | Missouri |
| Hawaii | North Carolina |
| Illinois | Oklahoma |
| Indiana*** | South Carolina |
| Iowa | South Dakota |
| Kentucky | Tennessee |
| Louisiana*** | Texas |
| Maryland | Utah |
| Michigan*** | Virginia |
| Minnesota** | Wyoming |
| Montana*** | |
| Nebraska | |
| Nevada | |
| New Hampshire*** | |
| New Jersey** | |
| New Mexico | |
| New York | |
| North Dakota | |
| Ohio | |
| Oregon | |
| Pennsylvania*** | |
| Rhode Island | |
| Vermont | |
| Washington** | |
| West Virginia | |

*Date of Medicaid Expansion was 1/1/2014 unless noted by ** or ***. Massachusetts and Wisconsin had prior Medicaid expansion independent of ACA passage, and were thus excluded from analyses.
**Early limited Medicaid expansion states
***Late Medicaid expansion states

**Supplemental Table 3. ICD-10 Codes for Cause-Specific Mortality**

| **Subcategory of Mortality** | **ICD-10 Codes** |
|---|---|
| External* Causes | U01-U03, V01-Y89 |
| Cardiovascular | I00-I99 |
| Respiratory | J00-J98 |
| Neoplasia | C00-D48 |
| Infection | A00-B99 |
| Prescription/Opioid Overdose | X40-X44, X60-X64, X85, Y10-Y14 |

*Internal causes includes all non-external causes of mortality

Centers for Disease Control and Prevention, National Center for Health Statistics. Underlying Cause of Death 1999-2018 on CDC WONDER Online Database, released in 2020. Data are from the Multiple Cause of Death Files, 1999-2018, as compiled from data provided by the 57 vital statistics jurisdictions through the Vital Statistics Cooperative Program. Accessed at http://wonder.cdc.gov/ucd-icd10.html on Dec 8, 2020.

**Supplemental Table 4. Evaluation of Parallel Trends Assumption for Difference-in-Differences Assessment between 2010-2013 for All-Cause and Cause-Specific Mortality**

| Cause of Mortality | Interaction Co-efficient* (95% CI) | Interaction p-value* |
|---|---|---|
| All Causes | -1.1 (-5.1 to +3.0) | 0.60 |
| Internal | -1.6 (-4.6 to +1.4) | 0.29 |
| External | +0.5 (-0.8 to +1.9) | 0.45 |
| Cardiovascular | -0.6 (-1.9 to +0.7) | 0.35 |
| Respiratory | -0.3 (-0.7 to +0.08) | 0.12 |
| Neoplasia | -0.1 (-0.8 to +0.6) | 0.77 |
| Infection | +0.05 (-0.3 to +0.4) | 0.73 |
| Prescription/Opioid Overdose | +0.4 (-0.07 to +0.9) | 0.09 |
| **All causes by state** | | |
| Alaska | +15.0 (-0.03 to +30.0) | 0.05 |
| Arizona | +4.0 (-5.5 to +13.4) | 0.39 |
| Arkansas | +12.7 (+2.6 to +22.8) | 0.02 |
| California | -11.4 (-22.2 to -0.6) | 0.04 |
| Colorado | +5.6 (-3.9 to +15.2) | 0.23 |
| Connecticut | +2.9 (-9.4 to +15.3) | 0.62 |
| Delaware | -0.1 (-11.8 to +11.6) | 0.98 |
| District of Columbia | -5.2 (-21.4 to +11.0) | 0.51 |
| Hawaii | +5.7 (-4.0 to +15.3) | 0.23 |
| Illinois | +3.4 (-6.0 to +12.8) | 0.46 |
| Indiana | -0.7 (-10.4 to +9.0) | 0.89 |
| Iowa | +8.5 (-2.1 to +19.1) | 0.11 |
| Kentucky | +5.2 (-3.7 to +14.2) | 0.23 |
| Louisiana | +4.1 (-5.3 to +13.5) | 0.37 |
| Maryland | +8.5 (-4.4 to +21.3) | 0.18 |
| Michigan | -16.0 (-28.9 to -3.1) | 0.02 |
| Minnesota | -0.3 (-10.4 to +9.8) | 0.95 |
| Montana | -3.2 (-17.9 to +11.4) | 0.65 |
| Nebraska | +9.7 (-2.4 to +21.8) | 0.11 |
| Nevada | -14.3 (-28.9 to +0.3) | 0.06 |
| New Hampshire | +4.5 (-10.0 to +19.0) | 0.52 |
| New Jersey | +3.3 (-8.2 to +14.8) | 0.56 |
| New Mexico | +8.9 (-1.3 to +19.0) | 0.08 |
| New York | +6.8 (-6.2 to +19.9) | 0.28 |
| North Dakota | +35.4 (+15.4 to +55.4) | 0.002 |
| Ohio | -2.8 (-12.9 to +7.4) | 0.57 |
| Oregon | -0.7 (-11.6 to +10.1) | 0.89 |
| Pennsylvania | +5.4 (-7.1 to +17.9) | 0.38 |
| Rhode Island | -0.6 (-13.0 to +11.9) | 0.92 |
| Vermont | +0.5 (-10.4 to +11.4) | 0.92 |
| Washington | -5.3 (-15.1 to +4.5) | 0.27 |
| West Virginia | +15.0 (+5.4 to +24.6) | 0.004 |

*Interaction between state expansion status and year as continuous variable between 2010-2013, adjusted for age strata, % female, % non-Hispanic Black, % Hispanic, % in poverty, % unemployed. P-values>0.05 indicate that the interaction between state expansion status and year were not significant between 2010-2013, thus suggesting parallel trends between expansion and non-expansion states before 2014.
States highlighted in Red (Alaska, Arkansas, California, Michigan, North Dakota, West Virginia) violate parallel trends assumption between 2010-2013 based on p<0.05 for the interaction indicating the parallel pre-trends assumption is violated. These states were not included in state-specific analyses.

**Supplemental Table 5. Evaluation of Parallel Trends Assumption for Difference-in-Differences Assessment between 2010-2013 for Cardiovascular-, Respiratory-, and Neoplasia- Mortality by State**

| | Cardiovascular | | Respiratory | | Neoplasia | |
|---|---|---|---|---|---|---|
| **State** | **Interaction Co-efficient* (95% CI)** | **p*** | **Interaction Co-efficient* (95% CI)** | **p*** | **Interaction Co-efficient* (95% CI)** | **p*** |
| Arizona | +0.03 (-3.6 to +3.7) | 0.99 | -0.6 (-1.2 to +0.06) | 0.07 | +1.2 (-0.6 to +3.0) | 0.17 |
| Colorado | +2.9 (-0.9 to +6.7) | 0.13 | -0.10 (-0.8 to +0.6) | 0.77 | +1.9 (+0.1 to +3.7) | 0.04 |
| Connecticut | +1.9 (-3.5 to +7.2) | 0.47 | +0.2 (-0.7 to +1.1) | 0.67 | +0.4 (-2.0 to +2.8) | 0.72 |
| Delaware | +1.7 (-3.4 to +6.8) | 0.48 | -0.5 (-1.4 to +0.4) | 0.23 | +0.3 (-2.0 to +2.5) | 0.79 |
| DC | +3.6 (-3.6 to +10.8) | 0.31 | +0.4 (-1.0 to +1.8) | 0.52 | +2.2 (-1.0 to +5.5) | 0.16 |
| Hawaii | +4.5 (+0.6 to +8.4) | 0.03 | +0.8 (+0.08 to +1.5) | 0.03 | +2.2 (+0.3 to +4.1) | 0.02 |
| Illinois | +1.8 (-1.9 to +5.5) | 0.32 | -0.3 (-0.9 to +0.4) | 0.42 | +1.2 (-0.6 to +3.0) | 0.18 |
| Indiana | -0.9 (-4.6 to +2.8) | 0.60 | -0.5 (-1.0 to +0.1) | 0.10 | -0.6 (-2.3 to +1.1) | 0.48 |
| Iowa | +4.0 (-0.1 to +8.2) | 0.06 | +0.9 (+0.2 to +1.7) | 0.02 | +2.8 (+0.9 to +4.8) | 0.006 |
| Kentucky | +1.4 (-2.1 to +4.9) | 0.42 | +0.3 (-0.3 to +0.8) | 0.33 | +0.9 (-0.7 to +2.6) | 0.26 |
| Louisiana | +0.7 (-3.1 to +4.5) | 0.70 | +0.4 (-0.2 to +1.1) | 0.19 | +1.3 (-0.5 to +3.2) | 0.14 |
| Maryland | +3.6 (-1.8 to +9.0) | 0.17 | +0.2 (-0.8 to +1.2) | 0.68 | +2.5 (-0.04 to +5.0) | 0.054 |
| Minnesota | +1.4 (-2.6 to +5.4) | 0.47 | +0.3 (-0.4 to +1.0) | 0.34 | +0.8 (-1.1 to +2.6) | 0.38 |
| Montana | -1.5 (-7.6 to +4.6) | 0.61 | -1.5 (-2.4 to -0.5) | 0.006 | +0.7 (-2.0 to +3.5) | 0.58 |
| Nebraska | +4.5 (-0.08 to +9.2) | 0.054 | +0.3 (-0.6 to +1.2) | 0.43 | +2.1 (-0.04 to +4.2) | 0.06 |
| Nevada | -6.3 (-11.1 to -1.5) | 0.01 | -1.0 (-2.1 to +0.03) | 0.06 | -3.5 (-5.8 to -1.2) | 0.006 |
| New Hampshire | +5.1 (-1.0 to +11.1) | 0.09 | -0.4 (-1.5 to +0.8) | 0.51 | +1.2 (-1.6 to +4.0) | 0.39 |
| New Jersey | +1.8 (-3.0 to +6.6) | 0.44 | +0.6 (-0.3 to +1.4) | 0.20 | -0.07 (-2.3 to +2.2) | 0.95 |
| New Mexico | +4.2 (+0.2 to +8.2) | 0.04 | +0.04 (-0.7 to +0.8) | 0.91 | +3.2 (+1.3 to +5.1) | 0.003 |
| New York | +3.2 (-2.3 to +8.8) | 0.24 | +0.2 (-0.8 to +1.3) | 0.64 | +1.8 (-0.8 to +4.4) | 0.16 |
| Ohio | -1.1 (-4.9 to +2.8) | 0.56 | -0.09 (-0.7 to +0.5) | 0.77 | +0.07 (-1.7 to +1.8) | 0.93 |
| Oregon | +0.2 (-3.9 to +4.4) | 0.92 | -0.6 (-1.2 to +0.02) | 0.06 | -0.4 (-2.4 to +1.5) | 0.64 |
| Pennsylvania | +3.3 (-1.8 to +8.4) | 0.19 | +0.6 (-0.4 to +1.5) | 0.24 | +1.1 (-1.4 to +3.5) | 0.37 |
| Rhode Island | +1.7 (-3.4 to +6.9) | 0.49 | -0.6 (-1.5 to +0.3) | 0.16 | +1.4 (-0.9 to +3.7) | 0.22 |
| Vermont | -0.3 (-4.7 to +4.1) | 0.88 | +1.0 (+0.2 to +1.9) | 0.02 | +1.5 (-0.6 to +3.6) | 0.16 |
| Washington | -2.0 (-5.7 to +1.6) | 0.26 | -0.6 (-1.2 to -0.04) | 0.04 | -0.5 (-2.2 to +1.2) | 0.53 |

*Interaction between state expansion status and year as continuous variable between 2010-2013, adjusted for age strata, % female, % non-Hispanic Black, % Hispanic, % in poverty, % unemployed. P-values>0.05 indicate that the interaction between state expansion status and year were not significant between 2010-2013, thus suggesting parallel trends between expansion and non-expansion states before 2014.

States highlighted in Red violate parallel trends assumption between 2010-2013 based on p<0.05 for the interaction indicating the parallel pre-trends assumption is violated. These states were not included in state-specific cause-specific mortality analyses.

**Supplemental Table 6. Evaluation of Parallel Trends Assumption for Difference-in-Differences Assessment between 2010-2013 for Infection- and Prescription/Opioid- Related Mortality by State**

| | Infection | | Prescription/Opioid | |
|---|---|---|---|---|
| **State** | **Interaction Co-efficient* (95% CI)** | **p*** | **Interaction Co-efficient* (95% CI)** | **p*** |
| Arizona | +0.1 (-0.2 to +0.5) | 0.36 | +0.4 (-0.5 to +0.8) | 0.08 |
| Colorado | +0.5 (+0.2 to +0.8) | 0.004 | +0.2 (-1.4 to +0.8) | 0.50 |
| Connecticut | +0.2 (-0.4 to +0.7) | 0.49 | +1.2 (+0.1 to +2.3) | 0.03 |
| Delaware | -0.9 (-1.4 to -0.5) | 0.001 | -0.6 (-1.6 to +0.3) | 0.18 |
| DC | -3.8 (-4.4 to -3.2) | <0.001 | -3.6 (-5.1 to -2.1) | <0.001 |
| Hawaii | +0.2 (-0.2 to +0.5) | 0.30 | -1.7 (-2.2 to -1.2) | <0.001 |
| Illinois | -0.2 (-0.5 to +0.07) | 0.13 | +0.5 (-0.1 to +1.2) | 0.12 |
| Indiana | +0.3 (+0.02 to +0.7) | 0.04 | +0.2 (-0.3 to +0.8) | 0.42 |
| Iowa | +0.7 (+0.3 to +1.1) | 0.001 | -0.8 (-1.7 to +0.2) | 0.10 |
| Kentucky | +0.8 (+0.5 to +1.0) | <0.001 | +1.4 (+1.0 to +1.9) | <0.001 |
| Louisiana | +0.6 (+0.2 to +0.9) | 0.002 | +0.4 (-0.1 to +0.9) | 0.11 |
| Maryland | +0.03 (-0.6 to +0.6) | 0.92 | +0.6 (-0.6 to +1.7) | 0.29 |
| Minnesota | +0.4 (+0.08 to +0.7) | 0.02 | +0.2 (-0.5 to +0.8) | 0.62 |
| Montana | -0.4 (-1.0 to +0.2) | 0.20 | +0.06 (-1.4 to +1.5) | 0.93 |
| Nebraska | +1.1 (+0.6 to +1.6) | <0.001 | -1.3 (-2.5 to -0.1) | 0.03 |
| Nevada | -0.8 (-1.5 to -0.04) | 0.04 | +1.2 (+0.2 to +2.4) | 0.03 |
| New Hampshire | +0.5 (-0.2 to +1.2) | 0.17 | -0.8 (-2.2 to +0.6) | 0.25 |
| New Jersey | -0.5 (-1.0 to -0.04) | 0.04 | +0.7 (-0.2 to +1.6) | 0.14 |
| New Mexico | +0.4 (+0.02 to +0.8) | 0.04 | -0.1 (-0.9 to +0.6) | 0.71 |
| New York | -0.2 (-0.8 to +0.4) | 0.48 | +0.09 (-1.1 to +1.3) | 0.88 |
| Ohio | +0.4 (+0.04 to +0.8) | 0.03 | +2.5 (+1.9 to +3.1) | <0.001 |
| Oregon | +0.5 (+0.06 to +1.0) | 0.03 | -1.0 (-1.9 to -0.1) | 0.03 |
| Pennsylvania | +0.3 (-0.3 to +0.8) | 0.30 | -0.4 (-1.4 to +0.7) | 0.46 |
| Rhode Island | -1.2 (-1.7 to -0.6) | <0.001 | +2.9 (+2.0 to +3.9) | <0.001 |
| Vermont | +0.4 (-0.01 to +0.8) | 0.06 | +1.2 (+0.5 to +1.9) | 0.003 |
| Washington | -0.2 (-0.6 to +0.2) | 0.25 | +0.2 (-0.3 to +0.7) | 0.41 |

*Interaction between state expansion status and year as continuous variable between 2010-2013, adjusted for age strata, % female, % non-Hispanic Black, % Hispanic, % in poverty, % unemployed. P-values>0.05 indicate that the interaction between state expansion status and year were not significant between 2010-2013, thus suggesting parallel trends between expansion and non-expansion states before 2014.

States highlighted in Red violate parallel trends assumption between 2010-2013 based on p<0.05 for the interaction indicating the parallel pre-trends assumption is violated. These states were not included in state-specific cause-specific mortality analyses.

**Supplemental Table 7. State-Specific Difference-in-Differences Estimates and Interrupted Time Series Analysis for All-Cause Mortality**

| State | DID Coefficient | Conley and Taber 95% CI | | ITSA Coefficient | 95% CI | |
|---|---|---|---|---|---|---|
| Delaware | -63.78 | -134.07 | -42.93 | -41.12 | -64.63 | -17.61 |
| Rhode Island | -54.22 | -124.25 | -33.24 | -29.17 | -55.09 | -3.25 |
| Connecticut | -47.87 | -116.57 | -26.95 | -26.17 | -49.70 | -2.65 |
| New York | -38.19 | -100.38 | -18.2 | -27.38 | -52.35 | -2.40 |
| New Jersey | -39.48 | -108.34 | -18.72 | -31.70 | -59.68 | -3.71 |
| Arizona | 15.62 | -56.51 | 35.92 | -10.42 | -37.66 | 16.82 |
| New Mexico | 30.37 | -39.79 | 51.38 | 34.14 | 10.39 | 57.88 |
| Pennsylvania | -27.86 | -93.71 | -7.28 | -19.76 | -42.95 | 3.44 |
| Louisiana | -19.24 | -88.84 | 1.55 | 1.61 | -34.92 | 38.15 |
| Maryland | -17.25 | -84.6 | 3.4 | -16.86 | -39.35 | 5.63 |
| Kentucky | 15.18 | -56.24 | 36.18 | -6.69 | -28.78 | 15.40 |
| Minnesota | -18.24 | -87.65 | 2.82 | -17.09 | -42.82 | 8.64 |
| Oregon | 19.89 | -51.22 | 40.27 | 4.36 | -25.26 | 33.98 |
| DC | 16.34 | -54.45 | 35.55 | 35.36 | 6.08 | 64.63 |
| Vermont | -17.56 | -87.84 | 3.67 | -4.43 | -33.08 | 24.22 |
| Nevada | -18.77 | -90.03 | 2.19 | -7.66 | -30.09 | 14.78 |
| Iowa | -10.64 | -80.87 | 10.32 | -13.88 | -35.56 | 7.80 |
| Illinois | -10.13 | -78.54 | 10.47 | -14.55 | -41.21 | 12.11 |
| Nebraska | 13.4 | -56.84 | 34.37 | 10.59 | -9.13 | 30.31 |
| Hawaii | -3.42 | -73.48 | 17.49 | -7.36 | -27.32 | 12.60 |
| New Hampshire | -18.98 | -88.33 | 1.95 | 14.37 | -15.02 | 43.77 |
| Washington | -3.47 | -74.86 | 17.45 | 8.60 | -10.82 | 28.02 |
| Ohio | 2.3 | -71.58 | 23.98 | -5.43 | -36.63 | 25.76 |
| Indiana | 2.61 | -68.62 | 23.55 | -7.84 | -27.86 | 12.18 |
| Montana | 2.34 | -68.37 | 23.32 | 9.52 | -22.16 | 41.18 |
| Colorado | -0.54 | -71.35 | 20.18 | -14.05 | -38.60 | 10.50 |

**Supplemental Table 8. State Characteristics Associated with Reduction in Difference-in-Differences for All-Cause Mortality Associated with Medicaid Expansion (Linear Regression)**

| Characteristic | Coef* (95% CI) |
|---|---|
| Women, per % | -17.8<br>(-26.7 to -8.8) |
| Non-Hispanic Black, per % | -1.4<br>(-2.4 to -0.3) |
| Hispanic, per % | +0.3<br>(-0.6 to +1.2) |
| Lives in Rural County, per % | +0.3<br>(-0.2 to +0.9) |
| Reduction in uninsured (2014-2018), per % | -5.4<br>(-9.0 to -1.8) |

*Adjusted for baseline age-adjusted mortality rate of the state at the beginning of the expansion era (2014). Coefficient corresponds to difference-in-difference for all-cause mortality associated with Medicaid expansion for each 2014 state characteristic, per unit of the characteristic, in number of deaths per 100,000 residents per year after Medicaid expansion. Negative values correspond to fewer deaths, and positive values correspond to an increase in deaths.

**Supplemental Table 9. Sensitivity Analysis 1: County-Level Difference-in-Differences Analysis for Subcategories of Mortality**

| Cause of Mortality | Absolute Difference* in Death Rate After Medicaid Expansion per 100,000 residents per year (95% CI) |
|---|---|
| All Causes | -6.5 (-14.6 to +1.5) |
| Internal | -8.1 (-15.2 to -0.9) |
| External | +1.5 (-4.2 to +7.2) |

*Difference-in-Differences analysis, which adjusted for age strata, % female, % non-Hispanic Black, % Hispanic, % in poverty, % unemployed, and the before-after trends in non-expansion states.

Cardiovascular, respiratory, neoplasia, infection, and prescription/opioid-related mortality not performed for county-level sensitivity analysis as more than 50% of counties were suppressed in those subcategories due to low number of events.

**Supplemental Table 10. Sensitivity Analysis 2: Difference-in-Differences Analysis for Subcategories of Mortality, Excluding Early (N=6) Adopters of Medicaid Expansion**

| **Cause of Mortality** | **Absolute Difference* in Death Rate After Medicaid Expansion per 100,000 residents per year (95% CI)** |
|---|---|
| All Causes | -8.4 (-21.1 to +4.3) |
| Internal | -9.9 (-18.3 to -1.4) |
| External | +1.5 (-12.7 to +3.5) |
| Cardiovascular | -4.6 (-7.8 to -1.5) |
| Respiratory | -1.3 (-2.5 to -0.1) |
| Neoplasia | -0.4 (-2.2 to +1.4) |
| Infection | -0.03 (-1.3 to +1.3) |
| Prescription/Opioid Overdose | +4.6 (+0.06 to +9.1) |

*Difference-in-Differences analysis, which adjusted for age strata, % female, % non-Hispanic Black, % Hispanic, % in poverty, % unemployed, and the before-after trends in non-expansion states.

**Supplemental Table 11. Sensitivity Analysis 2: Difference-in-Differences Analysis for Subcategories of Mortality, Excluding Late (N=7) Adopters of Medicaid Expansion**

| **Cause of Mortality** | **Absolute Difference* in Death Rate After Medicaid Expansion per 100,000 residents per year (95% CI)** |
|---|---|
| All Causes | -13.1 (-23.8 to -2.5) |
| Internal | -12.0 (-19.5 to -4.3) |
| External | -1.2 (-7.0 to +4.6) |
| Cardiovascular | -5.6 (-8.2 to -3.0) |
| Respiratory | -1.8 (-3.0 to -0.7) |
| Neoplasia | -1.0 (-2.8 to +0.9) |
| Infection | -0.3 (-1.4 to +0.8) |
| Prescription/Opioid Overdose | +2.3 (-2.0 to +6.5) |

*Difference-in-Differences analysis, which adjusted for age strata, % female, % non-Hispanic Black, % Hispanic, % in poverty, % unemployed, and the before-after trends in non-expansion states.

**Supplemental Table 12. Sensitivity Analysis 3: Difference-in-Differences Analysis for Subcategories of Mortality, Adjusting for Proportion of Population without Health Insurance**

| Cause of Mortality | Absolute Difference* in Death Rate After Medicaid Expansion per 100,000 residents per year (95% CI) |
|---|---|
| All Causes | -7.3 (-18.7 to +4.2) |
| Internal | -9.1 (-16.6 to -1.6) |
| External | +1.8 (-4.0 to +7.6) |
| Cardiovascular | -5.0 (-8.0 to -2.0) |
| Respiratory | -1.3 (-2.6 to -0.07) |
| Neoplasia | -0.2 (-2.1 to +1.6) |
| Infection | +0.6 (-0.6 to +1.8) |
| Prescription/Opioid Overdose | +3.6 (-0.5 to +7.7) |

*Difference-in-Differences analysis, which adjusted for age strata, % female, % non-Hispanic Black, % Hispanic, % in poverty, % unemployed, % population without health insurance, and the before-after trends in non-expansion states.

**Supplemental Table 13. Sensitivity Analysis 4: Difference-in-Differences Analysis for Subcategories of Mortality, Adjusting for Median State Income**

| Cause of Mortality | Absolute Difference* in Death Rate After Medicaid Expansion per 100,000 residents per year (95% CI) |
|---|---|
| All Causes | -5.7 (-15.8 to +4.4) |
| Internal | -7.4 (-14.0 to -0.8) |
| External | +1.6 (-4.0 to +7.2) |
| Cardiovascular | -4.1 (-7.0 to -1.2) |
| Respiratory | -1.1 (-2.2 to +0.07) |
| Neoplasia | -0.3 (-1.1 to +1.6) |
| Infection | -0.02 (-1.1 to +1.0) |
| Prescription/Opioid Overdose | +2.9 (-1.0 to +6.8) |

*Difference-in-Differences analysis, which adjusted for age strata, % female, % non-Hispanic Black, % Hispanic, % in poverty, % unemployed, median state income, and the before-after trends in non-expansion states.

**Supplementary Figure Legend**

**Supplementary Figure 1. Cohort Flowchart**
This figure details the flowchart that led to the primary cohort by expansion vs. non-expansion states for analysis.

**Supplementary Figure 2. Fully-Adjusted Mortality in Expansion vs. Non-Expansion States for Internal Causes of Death**
**Supplementary Figure 3. Fully-Adjusted Mortality in Expansion vs. Non-Expansion States for External Causes of Death**
**Supplementary Figure 4. Fully-Adjusted Mortality in Expansion vs. Non-Expansion States for Cardiovascular-Related Mortality**
**Supplementary Figure 5. Fully-Adjusted Mortality in Expansion vs. Non-Expansion States for Respiratory-Related Mortality**
**Supplementary Figure 6. Fully-Adjusted Mortality in Expansion vs. Non-Expansion States for Neoplasia-Related Mortality**
**Supplementary Figure 7. Fully-Adjusted Mortality in Expansion vs. Non-Expansion States for Infection-Related Mortality**
**Supplementary Figure 8. Fully-Adjusted Mortality in Expansion vs. Non-Expansion States for Prescription/Opioid Overdose Mortality**

Supplementary figures 1-7 represent cause-specific mortality rates by year, adjusted for age strata, % female, % non-Hispanic Black, % Hispanic, % in poverty, % unemployed, among expansion (yellow) and non-expansion (grey) states.

**Supplemental Figure 9. Post-Expansion Difference-in-Differences in Cardiovascular-Related Mortality among Expansion States**
**Supplemental Figure 10. Post-Expansion Difference-in-Differences in Respiratory-Related Mortality among Expansion States**
**Supplemental Figure 11. Post-Expansion Difference-in-Differences in Neoplasia-Related Mortality among Expansion States**
**Supplemental Figure 12. Post-Expansion Difference-in-Differences in Infection-Related Mortality among Expansion States**
**Supplemental Figure 13. Post-Expansion Difference-in-Differences in Prescription/Opioid Overdose-Related Mortality among Expansion States**

In these forest plots, the adjusted absolute difference in cause-specific mortality (blue dots) per 100,000 residents after Medicaid expansion with Conley and Taber 95% confidence intervals (error bars) obtained through multivariable difference-in-differences analysis, are presented for each expansion state meeting parallel trends assumption (Supplemental Tables 5-6). Negative values represent reductions in cause-specific mortality, whereas positive values represent increases in cause-specific mortality. State-specific difference-in-differences were estimated separately for each expansion state compared to the sample of non-expansion states. New Hampshire and New York for cardiovascular-related mortality, New Hampshire and Nevada for respiratory-related mortality, Pennsylvania and New York for neoplasia-related mortality did not meet the Holm step-down threshold for statistical significance.

**Supplementary Figure 1. Cohort Flowchart**

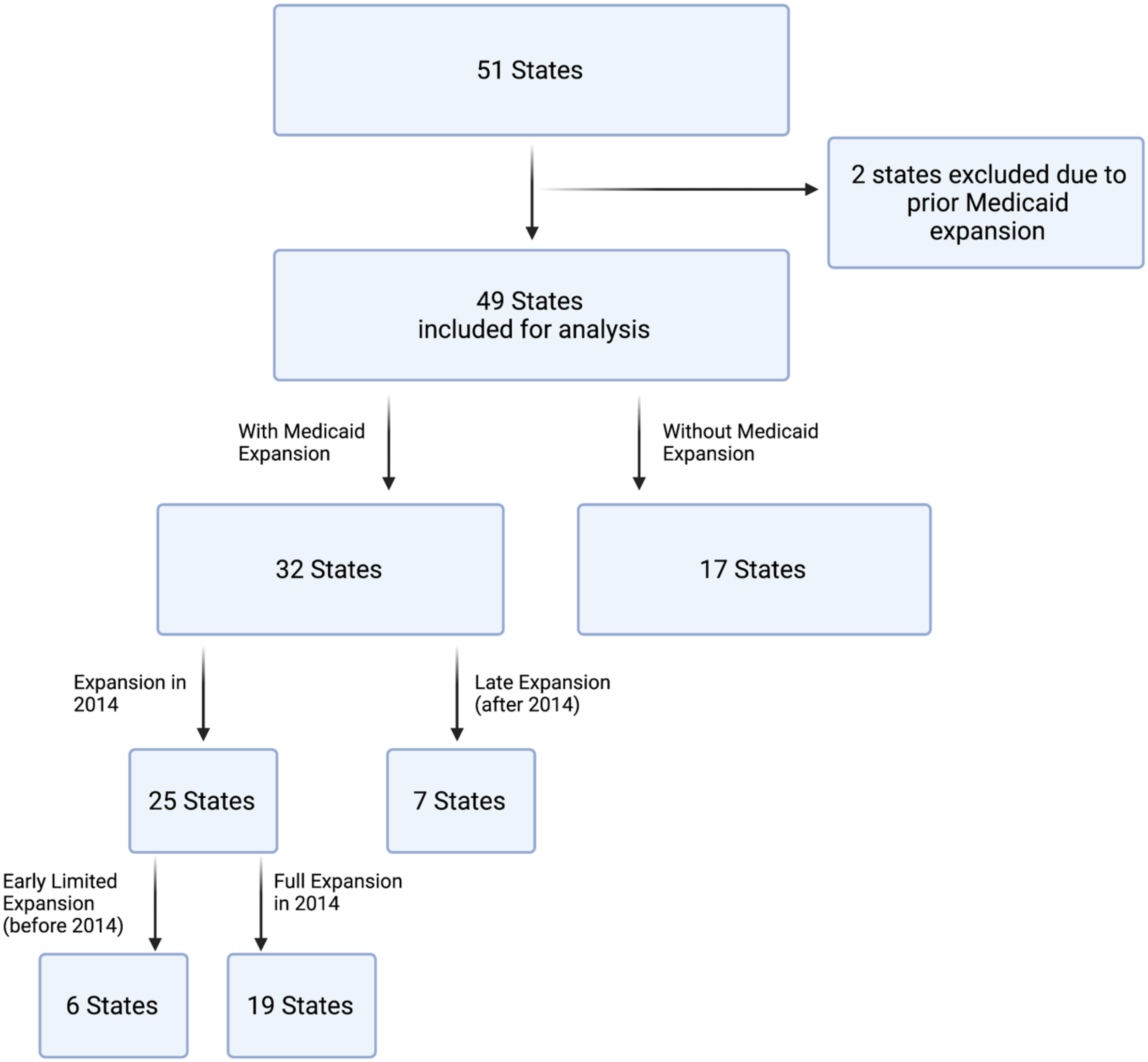

**Supplementary Figure 2. Fully-Adjusted Mortality in Expansion vs. Non-Expansion States for Internal Causes of Death**

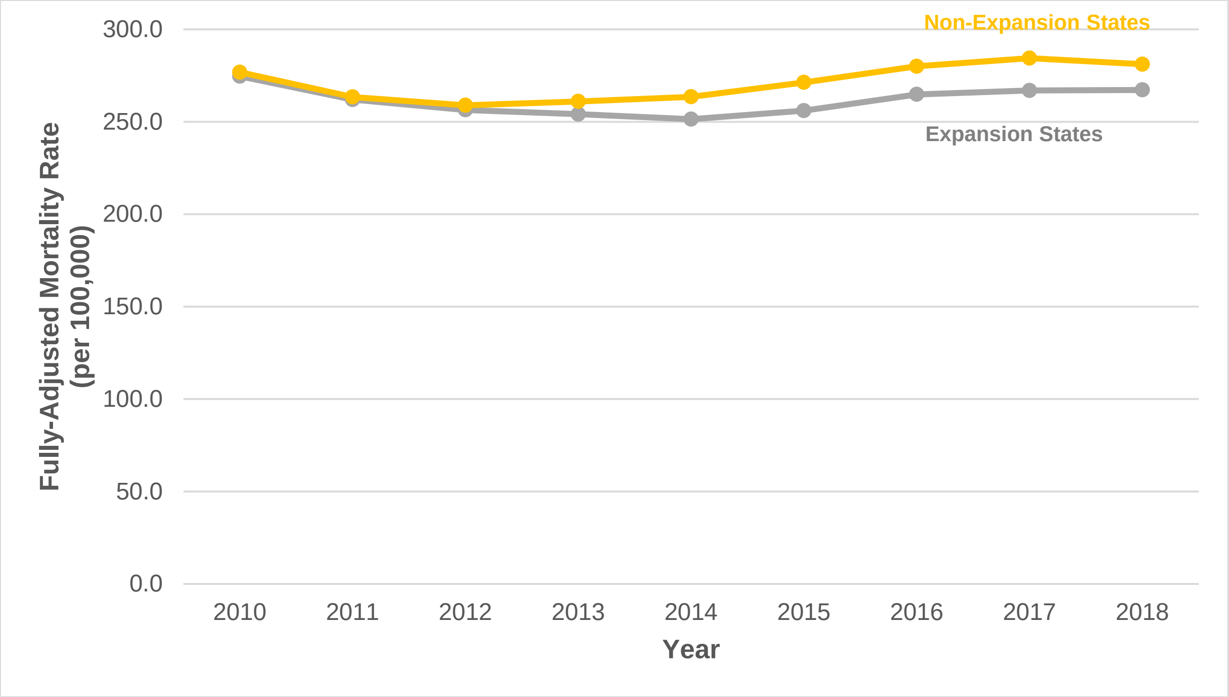


**Supplementary Figure 3. Fully-Adjusted Mortality in Expansion vs. Non-Expansion States for External Causes of Death**

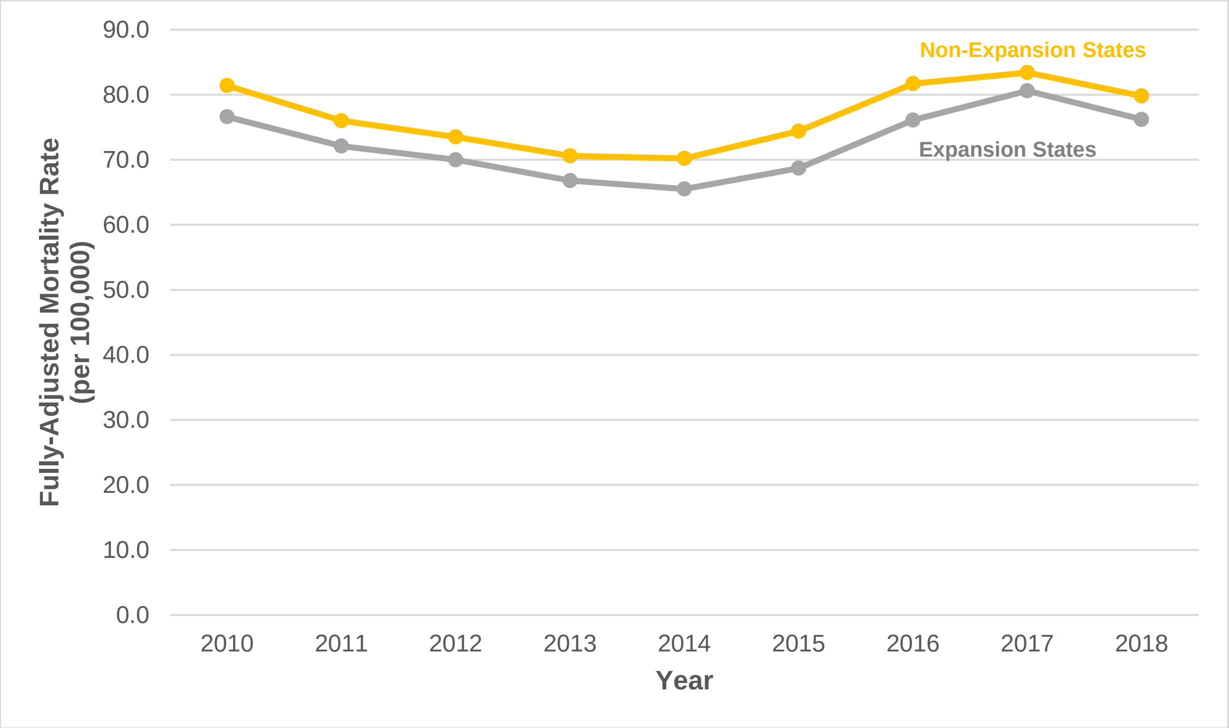

**Supplementary Figure 4. Fully-Adjusted Mortality in Expansion vs. Non-Expansion States for Cardiovascular-Related Mortality**

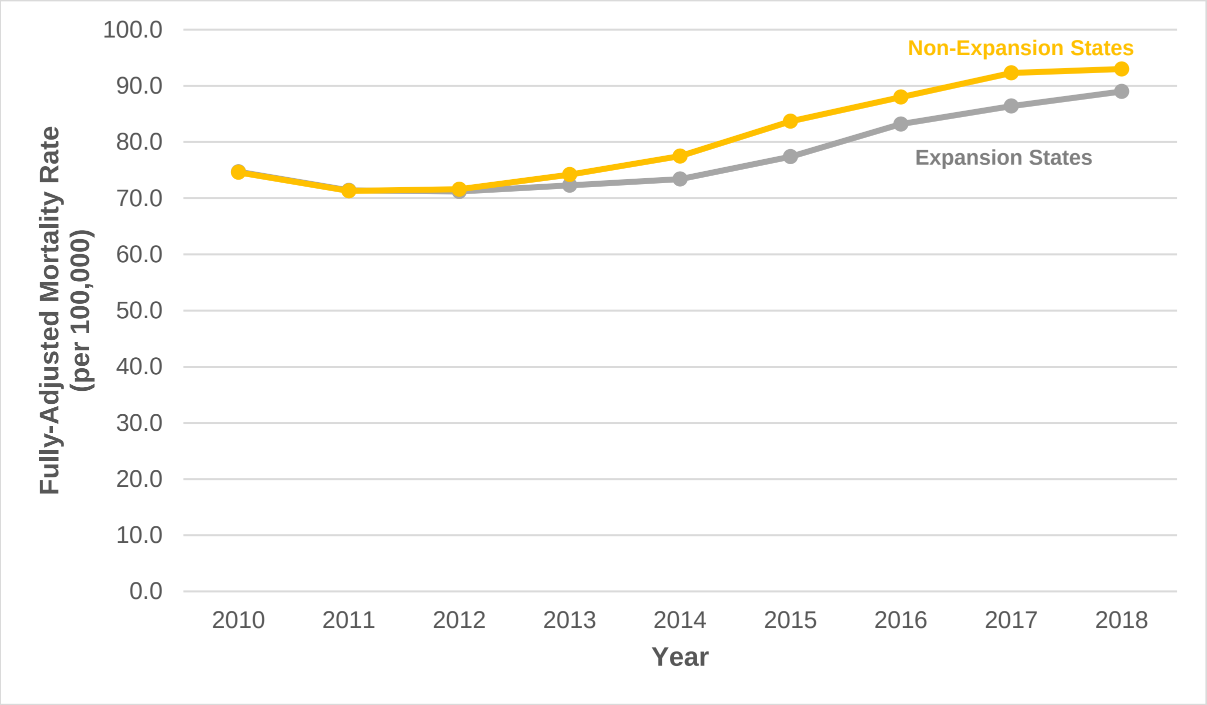


**Supplementary Figure 5. Fully-Adjusted Mortality in Expansion vs. Non-Expansion States for Respiratory-Related Mortality**

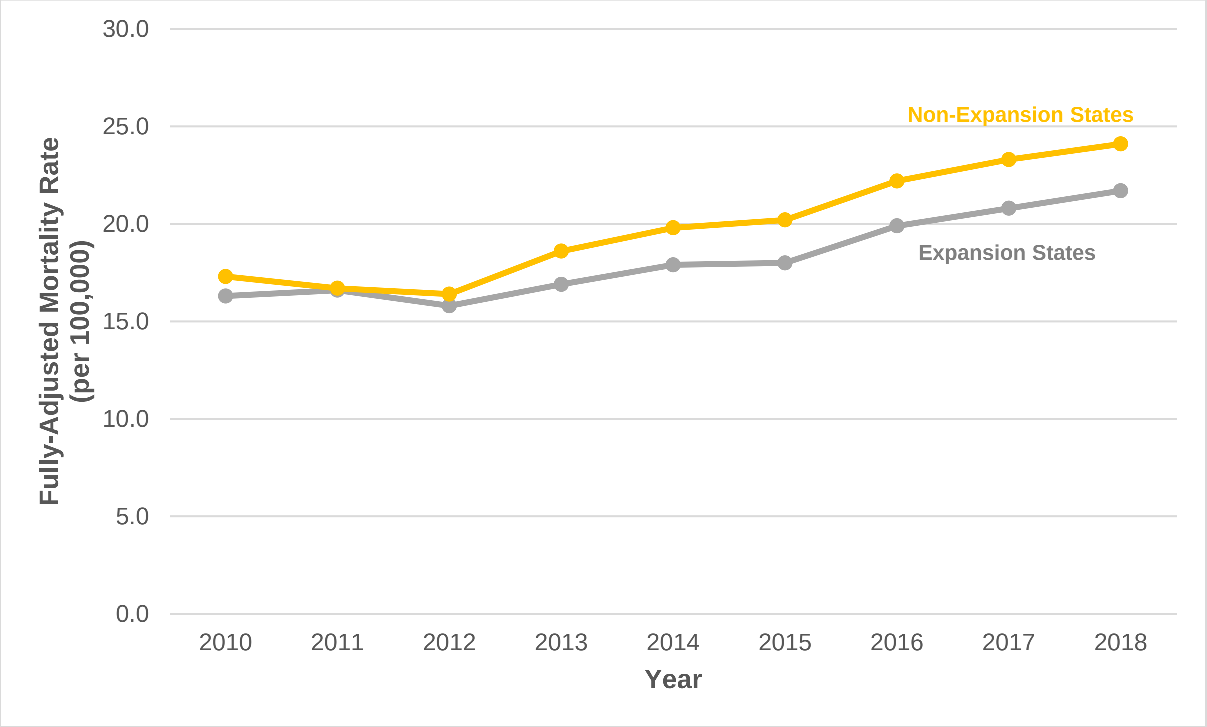

**Supplementary Figure 6. Fully-Adjusted Mortality in Expansion vs. Non-Expansion States for Neoplasia-Related Mortality**

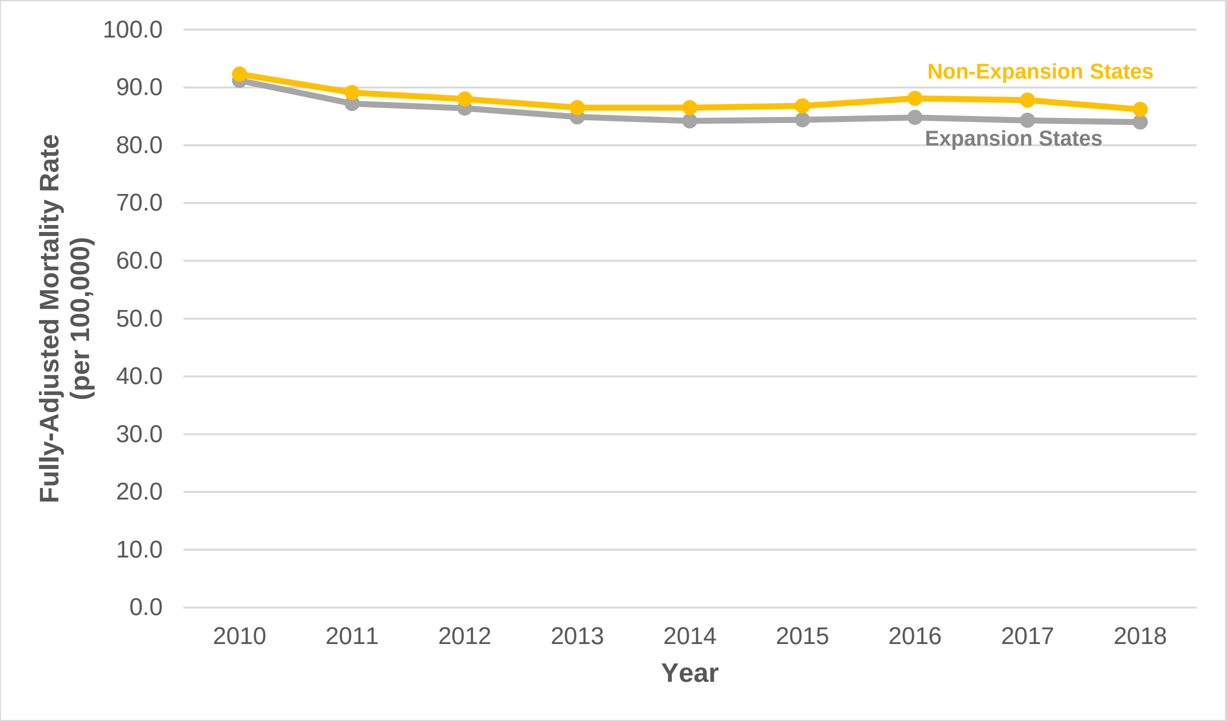


**Supplementary Figure 7. Fully-Adjusted Mortality in Expansion vs. Non-Expansion States for Infection-Related Mortality**

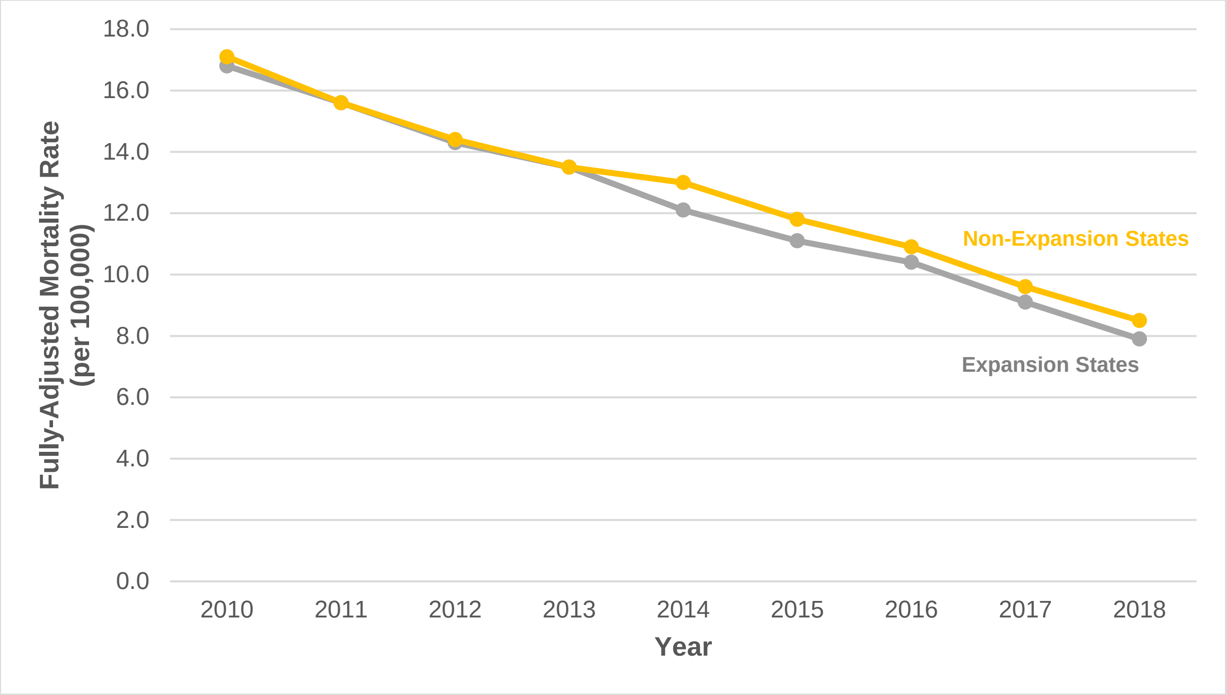

**Supplementary Figure 8. Fully-Adjusted Mortality in Expansion vs. Non-Expansion States for Prescription/Opioid Overdose Mortality**

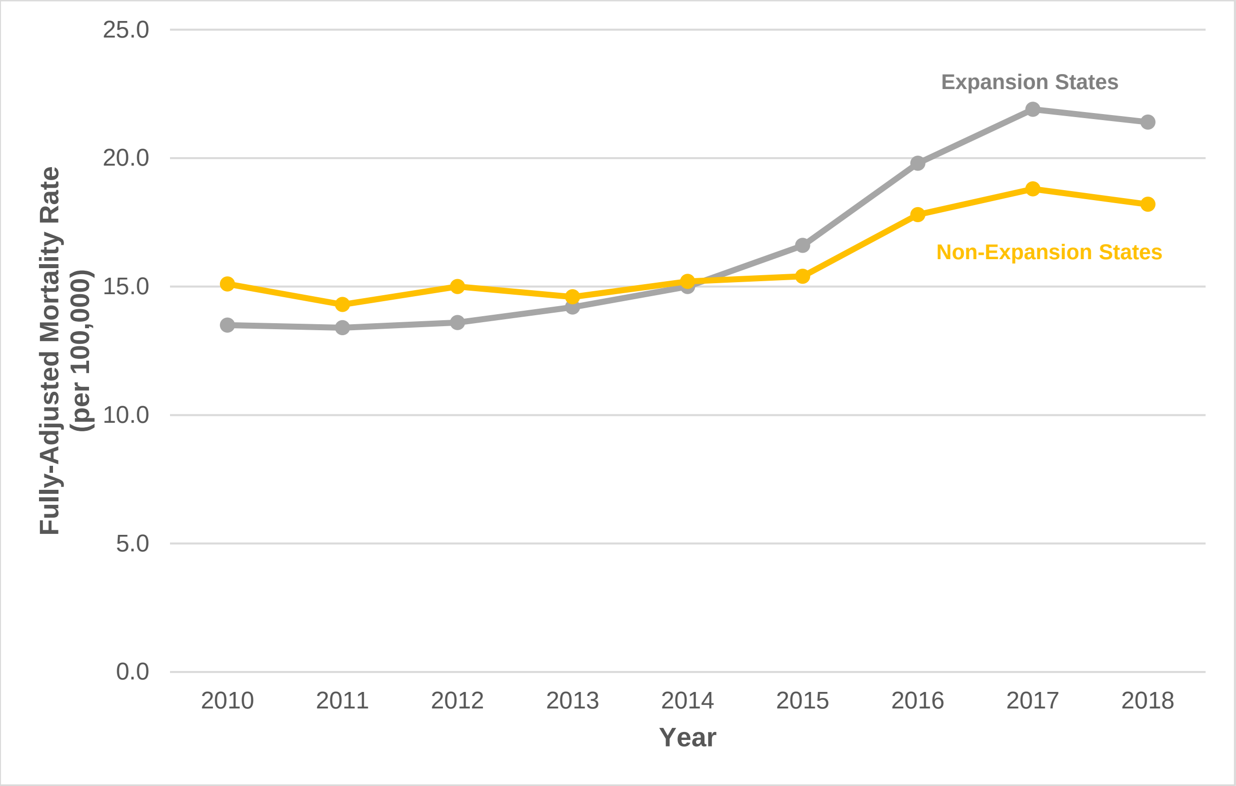

**Supplemental Figure 9. Post-Expansion Difference-in-Differences in Cardiovascular-Related Mortality among Expansion States**

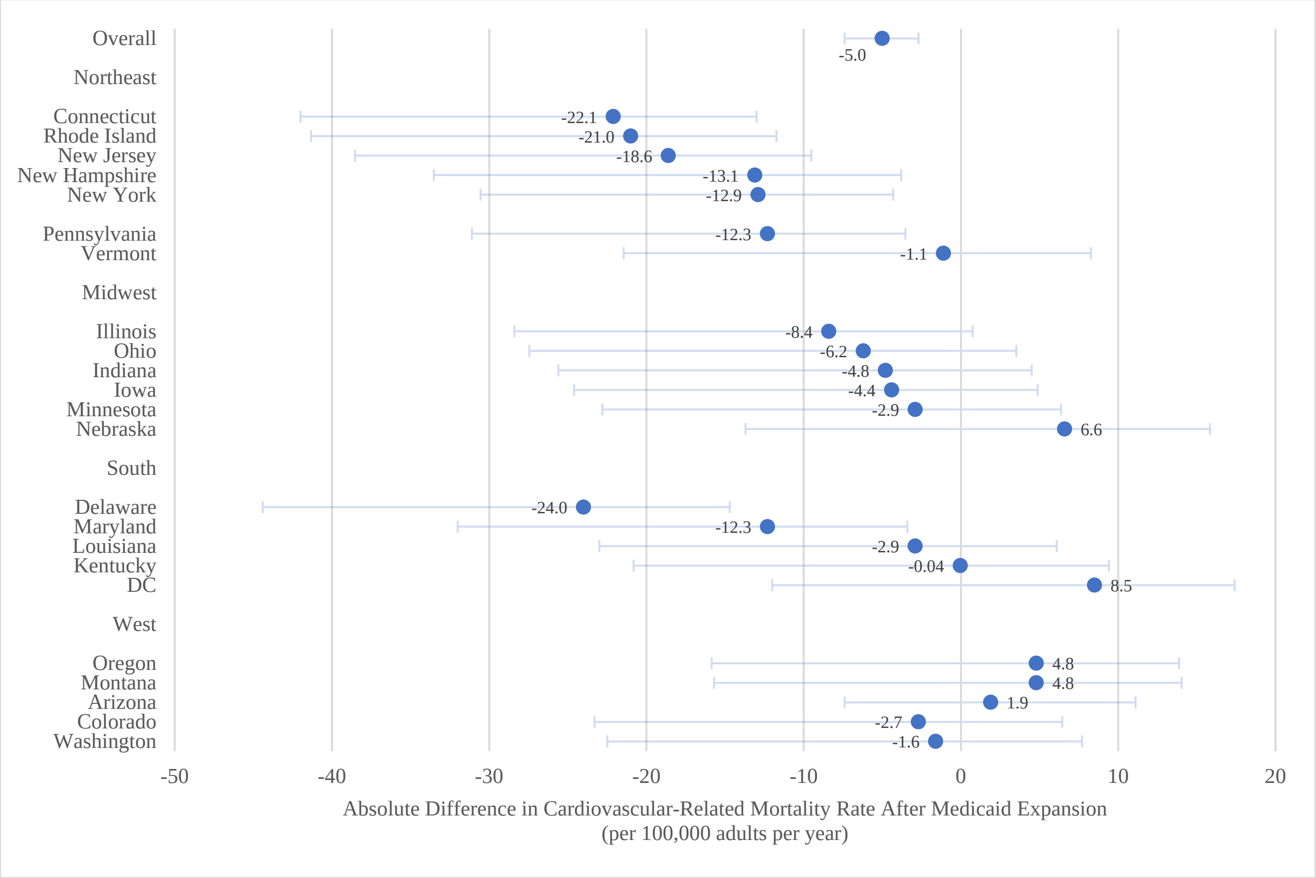


| US Census Region | State | Difference-in-Differences Coefficient | Conley and Taber 95% CI | |
|---|---|---|---|---|
| Northeast | Connecticut | -22.1 | -42.0 | -13.0 |
| | Rhode Island | -21.0 | -41.3 | -11.7 |
| | New Jersey | -18.6 | -38.5 | -9.5 |
| | New Hampshire | -13.1 | -33.5 | -3.8 |
| | New York | -12.9 | -30.6 | -4.3 |
| | Pennsylvania | -12.3 | -31.1 | -3.5 |
| | Vermont | -1.1 | -21.5 | 8.3 |
| Midwest | Illinois | -8.4 | -28.4 | 0.7 |
| | Ohio | -6.2 | -27.5 | 3.5 |
| | Indiana | -4.8 | -25.6 | 4.5 |
| | Iowa | -4.4 | -24.6 | 4.9 |
| | Minnesota | -2.9 | -22.8 | 6.4 |
| | Nebraska | 6.6 | -13.7 | 15.8 |
| South | Delaware | -24.0 | -44.4 | -14.7 |
| | Maryland | -12.3 | -32.0 | -3.4 |
| | Louisiana | -2.9 | -23.0 | 6.1 |
| | Kentucky | -0.04 | -20.8 | 9.4 |
| | DC | 8.5 | -12.0 | 17.4 |
| West | Oregon | 4.8 | -15.9 | 13.9 |
| | Montana | 4.8 | -15.7 | 14.0 |
| | Arizona | 1.9 | -7.4 | 11.1 |
| | Colorado | -2.7 | -23.3 | 6.4 |
| | Washington | -1.6 | -22.5 | 7.7 |
| Overall | | -5.0 | -7.4 | -2.7 |

**Supplemental Figure 10. Post-Expansion Difference-in-Differences in Respiratory-Related Mortality among Expansion States**

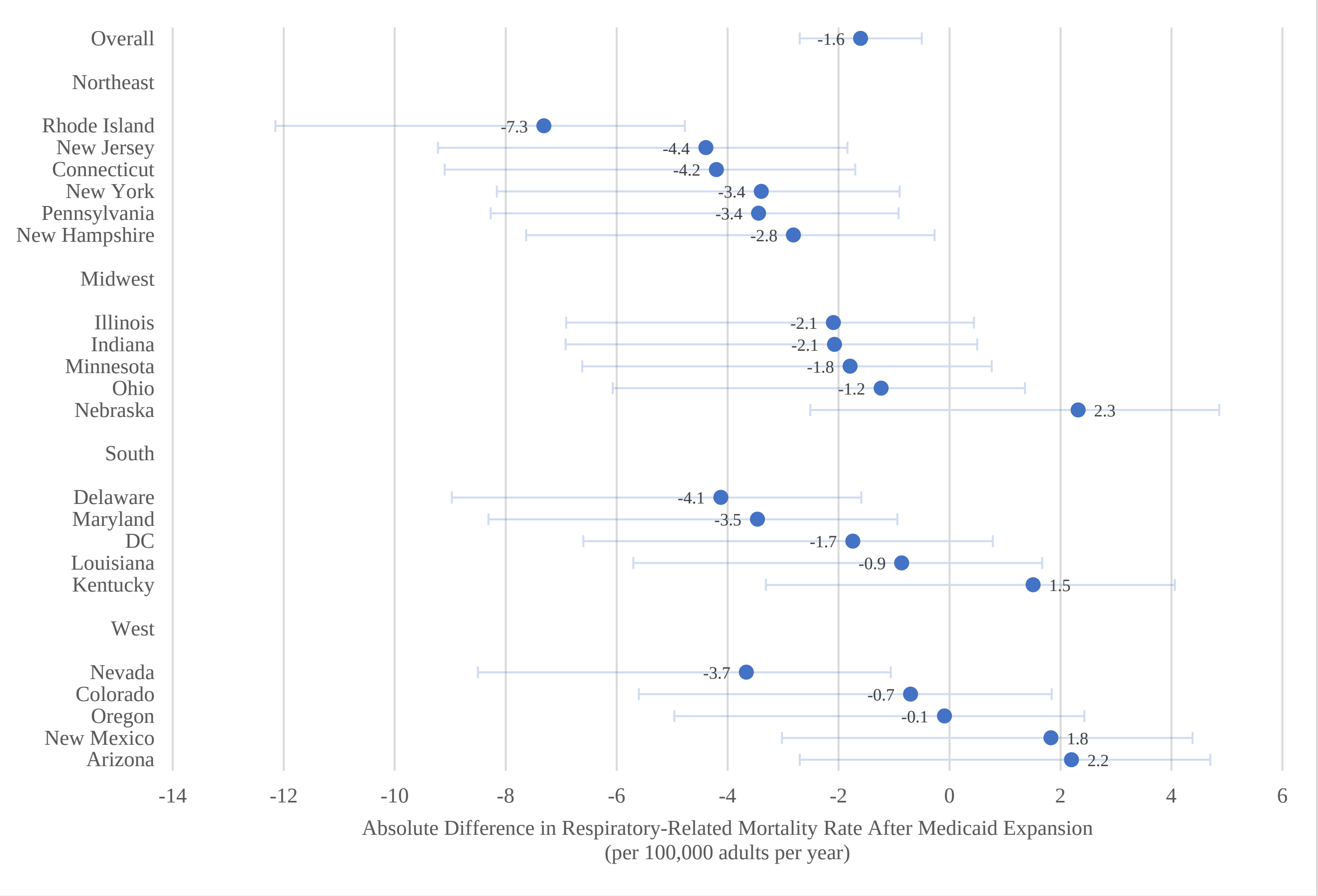


| US Census Region | State | Difference-in-Differences Coefficient | Conley and Taber 95% CI | |
|---|---|---|---|---|
| Northeast | Rhode Island | -7.3 | -12.2 | -4.8 |
| | New Jersey | -4.4 | -9.2 | -1.8 |
| | Connecticut | -4.2 | -9.1 | -1.7 |
| | New York | -3.4 | -8.2 | -0.9 |
| | Pennsylvania | -3.4 | -8.3 | -0.9 |
| | New Hampshire | -2.8 | -7.6 | -0.3 |
| Midwest | Illinois | -2.1 | -6.9 | 0.4 |
| | Indiana | -2.1 | -6.9 | 0.5 |
| | Minnesota | -1.8 | -6.6 | 0.8 |
| | Ohio | -1.2 | -6.1 | 1.4 |
| | Nebraska | 2.3 | -2.5 | 4.9 |
| South | Delaware | -4.1 | -9.0 | -1.6 |
| | Maryland | -3.5 | -8.3 | -0.9 |
| | DC | -1.7 | -6.6 | 0.8 |
| | Louisiana | -0.9 | -5.7 | 1.7 |
| | Kentucky | 1.5 | -3.3 | 4.1 |
| West | Nevada | -3.7 | -8.5 | -1.1 |
| | Colorado | -0.7 | -5.6 | 1.8 |
| | Oregon | -0.1 | -5.0 | 2.4 |
| | New Mexico | 1.8 | -3.0 | 4.4 |
| | Arizona | 2.2 | -2.7 | 4.7 |
| Overall | | -1.6 | -2.7 | -0.5 |

**Supplemental Figure 11. Post-Expansion Difference-in-Differences in Neoplasia-Related Mortality among Expansion States**

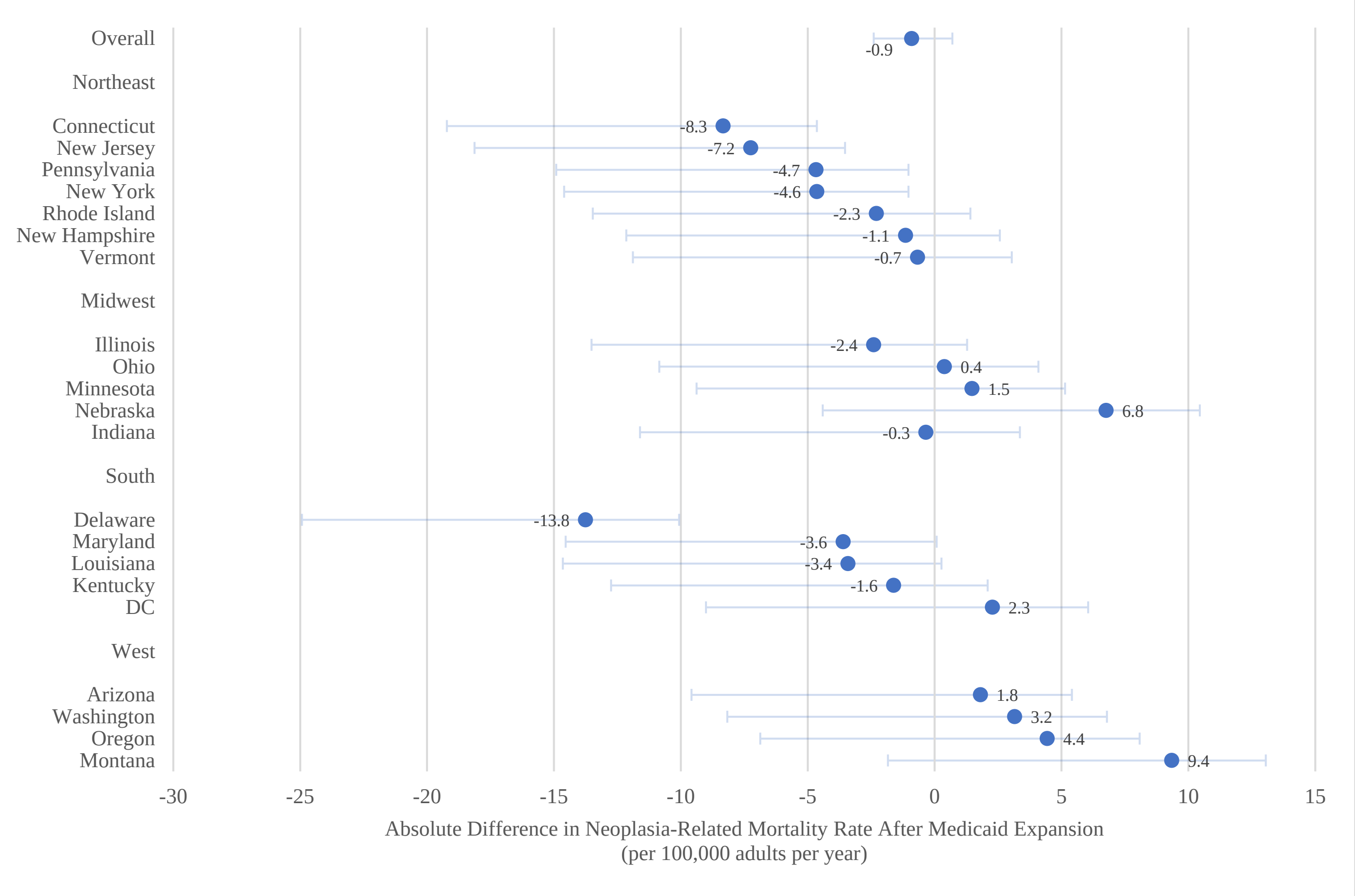


| US Census Region | State | Difference-in-Differences Coefficient | Conley and Taber 95% CI | |
|---|---|---|---|---|
| Northeast | Connecticut | -8.3 | -19.2 | -4.6 |
| | New Jersey | -7.2 | -18.1 | -3.5 |
| | Pennsylvania | -4.7 | -14.9 | -1.0 |
| | New York | -4.6 | -14.6 | -1.0 |
| | Rhode Island | -2.3 | -13.5 | 1.4 |
| | New Hampshire | -1.1 | -12.2 | 2.6 |
| | Vermont | -0.7 | -11.9 | 3.0 |
| Midwest | Illinois | -2.4 | -13.5 | 1.3 |
| | Ohio | 0.4 | -10.9 | 4.1 |
| | Minnesota | 1.5 | -9.4 | 5.1 |
| | Nebraska | 6.8 | -4.4 | 10.5 |
| | Indiana | -0.3 | -11.6 | 3.4 |
| South | Delaware | -13.8 | -24.9 | -10.1 |
| | Maryland | -3.6 | -14.5 | 0.1 |
| | Louisiana | -3.4 | -14.7 | 0.3 |
| | Kentucky | -1.6 | -12.8 | 2.1 |
| | DC | 2.3 | -9.0 | 6.1 |
| West | Arizona | 1.8 | -9.6 | 5.4 |
| | Washington | 3.2 | -8.2 | 6.8 |
| | Oregon | 4.4 | -6.9 | 8.1 |
| | Montana | 9.4 | -1.8 | 13.1 |
| Overall | | -0.9 | -2.4 | 0.7 |

**Supplemental Figure 12. Post-Expansion Difference-in-Differences in Infection-Related Mortality among Expansion States**

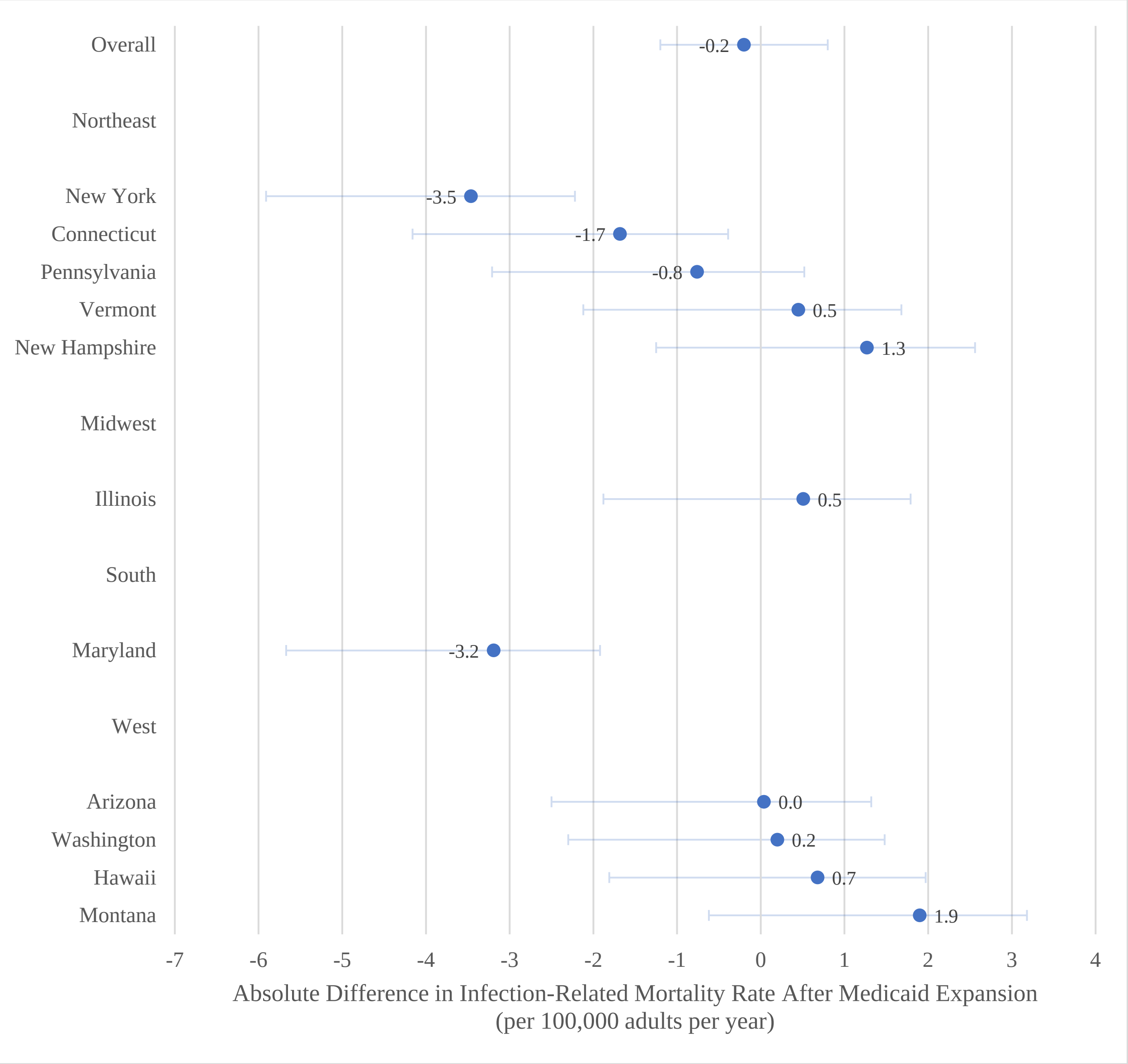


| US Census Region | State | Difference-in-Differences Coefficient | Conley and Taber 95% CI | |
|---|---|---|---|---|
| Northeast | New York | -3.5 | -5.9 | -2.2 |
| | Connecticut | -1.7 | -4.2 | -0.4 |
| | Pennsylvania | -0.8 | -3.2 | 0.5 |
| | Vermont | 0.5 | -2.1 | 1.7 |
| | New Hampshire | 1.3 | -1.3 | 2.6 |
| Midwest | Illinois | 0.5 | -1.9 | 1.8 |
| South | Maryland | -3.2 | -5.7 | -1.9 |
| West | Arizona | 0.0 | -2.5 | 1.3 |
| | Washington | 0.2 | -2.3 | 1.5 |
| | Hawaii | 0.7 | -1.8 | 2.0 |
| | Montana | 1.9 | -0.6 | 3.2 |
| Overall | | -0.2 | -1.2 | 0.8 |

**Supplemental Figure 13. Post-Expansion Difference-in-Differences in Prescription/Opioid-Related Mortality among Expansion States**

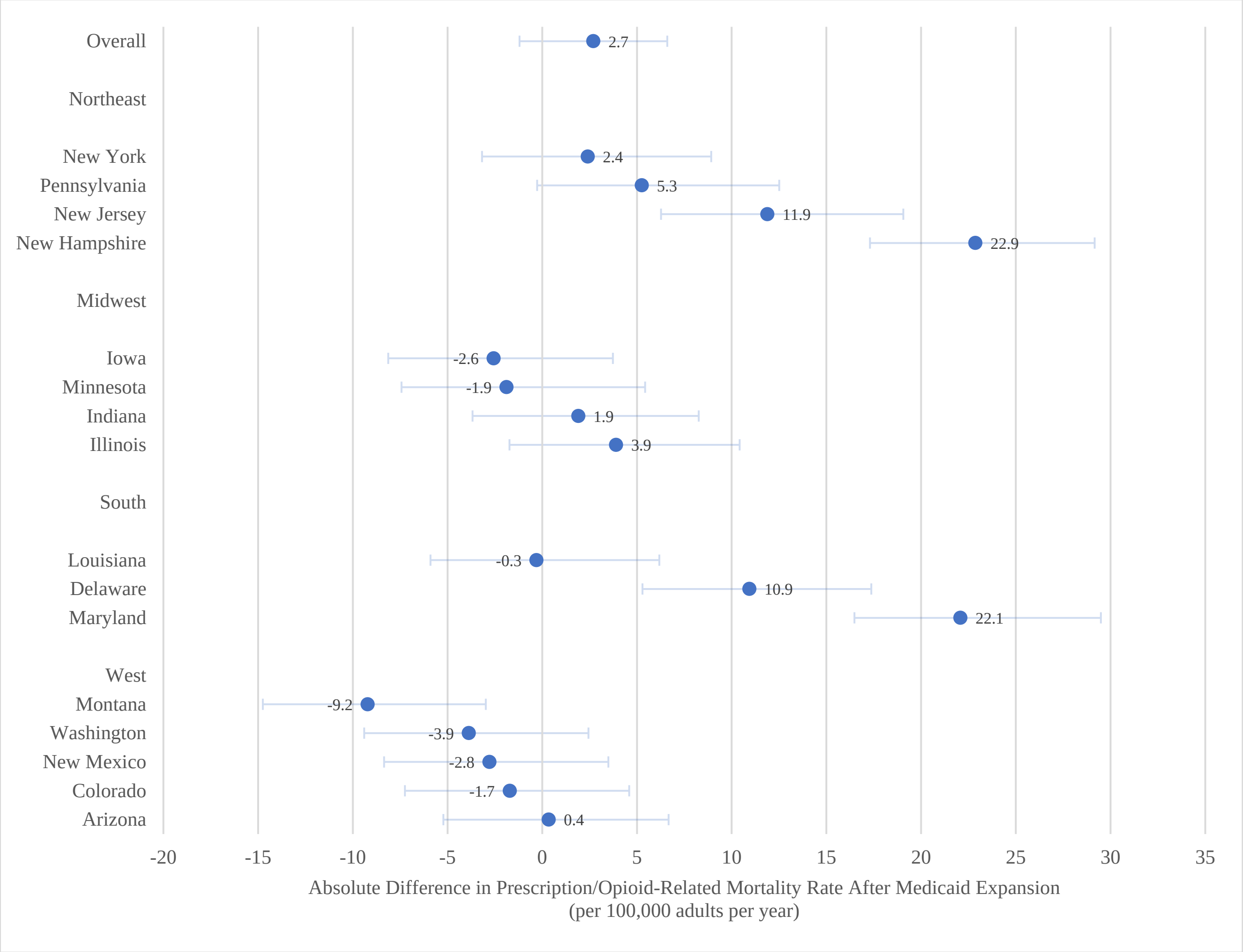


| US Census Region | State | Difference-in-Differences Coefficient | Conley and Taber 95% CI | |
|---|---|---|---|---|
| Northeast | New York | 2.4 | -3.2 | 8.9 |
| | Pennsylvania | 5.3 | -0.3 | 12.5 |
| | New Jersey | 11.9 | 6.3 | 19.1 |
| | New Hampshire | 22.9 | 17.3 | 29.2 |
| Midwest | Iowa | -2.6 | -8.1 | 3.7 |
| | Minnesota | -1.9 | -7.4 | 5.4 |
| | Indiana | 1.9 | -3.7 | 8.3 |
| | Illinois | 3.9 | -1.7 | 10.4 |
| South | Louisiana | -0.3 | -5.9 | 6.2 |
| | Delaware | 10.9 | 5.3 | 17.4 |
| | Maryland | 22.1 | 16.5 | 29.5 |
| West | Montana | -9.2 | -14.8 | -3.0 |
| | Washington | -3.9 | -9.4 | 2.4 |
| | New Mexico | -2.8 | -8.4 | 3.5 |
| | Colorado | -1.7 | -7.3 | 4.6 |
| | Arizona | 0.4 | -5.2 | 6.7 |
| Overall | | 2.7 | -1.2 | 6.6 |
